# Efficient PCA denoising of spatially correlated redundant MRI data

**DOI:** 10.1162/imag_a_00049

**Published:** 2023-12-18

**Authors:** Rafael Neto Henriques, Andrada Ianuş, Lisa Novello, Jorge Jovicich, Sune N Jespersen, Noam Shemesh

**Affiliations:** Champalimaud Research, Champalimaud Foundation, Lisbon, Portugal; Center for Mind/Brain Sciences - CIMeC, University of Trento, Rovereto, Italy; Center of Functionally Integrative Neuroscience (CFIN) and MINDLab, Clinical Institute, Aarhus University, Aarhus, Denmark; Department of Physics and Astronomy, Aarhus University, Aarhus, Denmark

**Keywords:** denoising, PCA, noise variance, diffusion MRI, DTI, DKI

## Abstract

Marčenko-Pastur PCA (MPPCA) denoising is emerging as an effective means for noise suppression in MR imaging (MRI) acquisitions with redundant dimensions. However, MPPCA performance can be severely compromised by spatially correlated noise—an issue typically affecting most modern MRI acquisitions—almost to the point of returning the original images with little or no noise removal. In this study, we explore different threshold criteria for principal component analysis (PCA) component classification that enable efficient and robust denoising of MRI data even when noise exhibits high spatial correlations, especially in cases where data are acquired with Partial Fourier and when only magnitude data are available. We show that efficient denoising can be achieved by incorporating a-priori information about the noise variance into PCA denoising thresholding. Based on this, two denoising strategies developed here are: 1) General PCA (GPCA) denoising that uses a-priori noise variance estimates without assuming specific noise distributions; and 2) Threshold PCA (TPCA) denoising which removes noise components with a threshold computed from a-priori estimated noise variance to determine the upper bound of the Marčenko-Pastur (MP) distribution. These strategies were tested in simulations with known ground truth and applied for denoising diffusion MRI data acquired using pre-clinical (16.4T) and clinical (3T) MRI scanners. In synthetic phantoms, MPPCA denoising failed to denoise spatially correlated data, while GPCA and TPCA better classified components as dominated by signal/noise. In cases where the noise variance was not accurately estimated (as can be the case in many practical scenarios), TPCA still provides excellent denoising performance. Our experiments in pre-clinical diffusion data with highly corrupted by spatial correlated noise revealed that both GPCA and TPCA robustly denoised the data while MPPCA denoising failed. In *in vivo* diffusion MRI data acquired on a clinical scanner in healthy subjects, MPPCA weakly removed noised, while TPCA was found to have the best performance, likely due to misestimations of the noise variance. Thus, our work shows that these novel denoising approaches can strongly benefit future pre-clinical and clinical MRI applications.

## 1 Introduction

The difference in Zeeman energy levels which endows Nuclear Magnetic Resonance (MR) with observable signals is inherently small, typically associated with frequencies in the MHz-GHz range. In MR imaging (MRI), these low-energy resonances ensure the safety of the methodology, as strong irradiation is not required to excite spins. However, due to the smallness of the ensuing signal, thermal noise in the receiver coils (Constantinides et al., 1997; Henkelman, 1985) is a significant component of detected data and effectively limits MRI’s spatiotemporal resolution. Furthermore, the potential usefulness of MRI contrasts is often signal-to-noise limited. For example, in diffusion MRI (dMRI) and relaxometry, the already inherently low signals are exacerbated by the need to attenuate the signal for contrast, leading to even more significant losses of spatiotemporal resolution. In functional MRI and methods based on image difference methods (e.g., Magnetization Transfer, Chemical exchange saturation transfer), the contrast to noise is low, and in Magnetic Resonance Spectroscopy (MRS), signals with five orders of magnitude smaller than that of the concentrated water protons are being sought. Thus, the suppression of thermal noise effects is of general interest in MRI.

Hardware improvements, such as more efficient coil designs, provide higher signal-to-noise ratio (SNR) from the coil (Kaza et al., 2011; Rodríguez & Medina, 2005; Roemer et al., 1990; Schmitt & Rieger, 2021). More recently, the introduction of cryogenic coils has shown how suppression of thermal noise by a factor of ~2-5 can produce improvements in image quality (Hall et al., 1991; Kwok & You, 2006; Niendorf et al., 2015; Poirier-Quinot et al., 2008); however, these coils are expensive and difficult to handle (Labbé et al., 2020, 2021). On the other hand, suppression of noise via image processing is a very active field of research, which can potentially provide significant gains in SNR, synergistically with improved coil designs. Several such techniques have been proposed, including standard image-domain smoothing and filtering (Friston et al., 1995; Jones & Cercignani, 2010; Jones et al., 2005), edge-preserving anisotropic filters (Gerig et al., 1992), wavelet transformations (Nowak, 1999; Pižurica et al., 2003), total variation minimization (Knoll et al., 2011), and non-local means (Coupé et al., 2008; Manjón et al., 2010, 2008). However, since these techniques rely on assumptions on spatial features of the data, such methods can compromise anatomical details by introducing spatial smoothing, blurring, staircase artifacts, and other types of image intensity bias (K. Kay, 2022; Tax et al., 2022; Veraart, Fieremans, Jelescu, et al., 2016; Veraart, Novikov, et al., 2016).

Denoising strategies based on Principal component analysis (PCA) had previously been used to provide a more optimal compromise between noise suppression and preservation of signal information by exploring signal redundancy (Deledalle et al., 2011; Hansen et al., 2014; Murali Mohan Babu et al., 2012). Redundancy exists in many types of MRI methods where larger amount of data samples are acquired relative to the relevant degrees of freedom. This is the case for diffusion MRI data that are acquired for, for example, different diffusion gradient directions, different b-values, and/or different diffusion times (Manjón et al., 2013, 2015; Pai et al., 2011; Veraart, Novikov, et al., 2016). In MRI relaxometry, signals are acquired for multiple echoes (Bazin et al., 2019; Does et al., 2019), and, in functional MRI or functional MRS (Adhikari et al., 2019; Mosso et al., 2022), redundancy arises from repeating the scans along the paradigm.

In such acquisitions, the denoising procedure is based on a reshaping of data as an M×N dimensional matrix, where M represents voxels (which can correspond to all image voxels or selected voxels according to a sliding window (Manjón et al., 2013; Veraart, Novikov, et al., 2016)) and N corresponds to the redundant dimension (e.g., along different diffusion gradient acquisitions, echoes, or functional MRI/MRS time points). Following a PCA, a relatively small number of principal components typically carry mainly signal information while the remaining components carry mainly noise (Manjón et al., 2013) (n.b., the signal components are also perturbed by the noise). In diffusion MRI, for example, it was shown that PCA eigenvalues mainly corresponding to noise can be removed using a threshold calculated based on an a-priori noise variance estimate (Pai et al., 2011), typically, in addition to empirically adjusted conversion factor according to different acquisition schemes (Manjón et al., 2013, 2015).

Later, the PCA component classification was objectified by Veraart, Fieremans, and Novikov (2016) and Veraart, Novikov, et al. (2016) using concepts from random matrix theory and assuming that PCA noise-related eigenvalues are characterized by a Marčenko-Pastur distribution (Marčenko & Pastur, 1967). The Marčenko-Pastur PCA (MPPCA) denoising has since become one of the most employed algorithms for diffusion MRI pre-processing. The MPPCA denoising has shown promising results not only for diffusion MRI (Ades-Aron et al., 2018; Gurney-Champion et al., 2019; Henriques et al., 2021; Moeller et al., 2021; Olesen et al., 2023; Pai et al., 2011; Shemesh, 2018; Tournier et al., 2019; Veraart, Novikov, et al., 2016), but also for other MRI modalities such as MRI relaxometry (Bazin et al., 2019; Does et al., 2019), functional MRI (Ades-Aron et al., 2020; Adhikari et al., 2019; Diao et al., 2021; Fernandes et al., 2022; Vizioli et al., 2021), MRS (Froeling et al., 2021; Simões et al., 2022), and functional MRS (Mosso et al., 2022). However, it is important to note that MPPCA denoising schemes assume that noise is identically distributed, spatially uniform, and spatially uncorrelated—which can be violated significantly in real-life data and lead to poor denoising performance (Cordero-Grande et al., 2019; Moeller et al., 2021).

In typical MRI acquisitions, noise is non-central distributed, spatially varying, and spatially correlated as a consequence of different reconstruction steps such as coil combination and parallel imaging (Aja-Fernández & Tristán-Vega, 2012; Aja-Fernández et al., 2011, 2014, 2015; Constantinides et al., 1997; Pruessmann et al., 1999), k-space gridding, zero-filling and partial Fourier acquisitions (Landman, Bazin, Smith, et al., 2009), spatial smoothing and interpolation during the image reconstruction (Jezzard & Balaban, 1995), and wrapped phase (Cordero-Grande et al., 2019; Moeller et al., 2021; Vizioli et al., 2021), among others. In many cases, data are obtained after multiple pre-processing steps, sometimes irreversibly (e.g., vendor reconstruction), and so these noise characteristics can be an important confounding factor for MPPCA denoising. To improve the performance of MPPCA denoising, recent studies suggested the use of additional pre-processing steps to ensure that noise is identically distributed, spatially uniform, and uncorrelated before denoising (Cordero-Grande et al., 2019; Moeller et al., 2021; Vizioli et al., 2021). These approaches used the information from complex data to ensure data reconstruction with zero-mean and identically distributed noise and to correct spatial variance and correlated noise.

Here, we focus on understanding how noise spatial correlations impact PCA denoising and how different threshold criteria for PCA component classification can still provide robust denoising performance, even in typical magnitude reconstructed data. Particularly, we show that a significant improvement in the classification of signal and noise components can be achieved by adding prior information on the noise variance. Based on this, two novel PCA denoising strategies are developed: 1) the General PCA (GPCA) denoising uses noise variance estimates determined a-priori without assuming specific noise distribution functions; and 2) the Threshold PCA (TPCA) denoising combines the noise variance prior estimate with MP distribution characteristics to define a threshold for noise component removal. We present the relevant theory, and demonstrate the advantages of these two novel denoising strategies in simulations where ground truth is known, and diffusion MRI data acquired using pre-clinical (16.4T) and a clinical (3T) scanners (all code used to produce the figures of this paper is freely available at https://github.com/RafaelNH/PCAdenoising).

## 2 Methods

### 2.1 Theory

#### 2.1.1 PCA denoising

Let us define an M  ×  N matrix X that contains the N redundant measurements (in the case of our dMRI data, these correspond to the different diffusion-weighted signals acquired for different gradient directions and b-values) for M neighboring voxels typically selected in a sliding window. Each column of X is subtracted by its mean. Without loss of generality, we consider here matrices with M  ≥  N, but the theory presented below can easily be rewritten for matrices with *M < N*. The principal component analysis of X can be performed from the eigen-decomposition of its covariance matrix:

$$\text{U}\Lambda \;{\text{U}}^{T}=\frac{1}{M}X^{T}X$$ (1)

where U is an N×N matrix that contains all PCA eigenvectors, and Λ is an N×N diagonal matrix containing the respective eigenvalues λ. PCA denoising can be achieved by excluding *C* components mainly associated with noise in the eigenvalue spectrum—the expected SNR gain depends on both N and *C* (SNR gain = $\sqrt{N/\left(N-C\right)}$, Veraart, Novikov, et al., 2016). In pioneering work using PCA to denoise MRI data (Manjón et al., 2013, 2015), this was performed by zeroing eigenvalues lower than a given threshold τ calculated by:

$$\tau =\upsilon^{2}{\hat{\sigma }}^{2}$$ (2)

where ${\hat{\sigma }}^{2}$ is the noise variance (typically estimated a-priori) and υ is an empirically defined correction factor (Manjón et al., 2013, 2015). Denoised signals for each sliding window are then reconstructed from the eigenvalues (and eigenvectors) surviving the thresholding in PCA space. Denoised datasets can then be fully reconstructed from the middle voxel of each sliding window, or by combining the denoised signals from overlapping voxels from adjacent sliding windows by using, for example, the overcomplete averaging procedure (Katkovnik et al., 2009; Manjón et al., 2013). Due to its previous reported advantages (Katkovnik et al., 2009), overcomplete averaging is used for all denoising strategies explored in this study.

Although the pioneering work mentioned above had already used a-prior noise variance estimates, latter studies proposed alternative strategies for component classification to avoid the use of the subjective correction factor (see section 2.1.2). In this study, we show that a-priori noise variance estimates can be used with PCA denoising without employing empirically defined correction factors (see sections 2.1.3 and 2.1.4).

#### 2.1.2 MPPCA denoising

According to Marčenko and Pastur (1967), a matrix X populated entirely by pure Gaussian random noise with variance ${\hat{\sigma }}^{2}$ in the limit of ${\left(M,N\right)}_{\to \infty }$, with γ=N/M fixed, propagates to the PCA eigenvalues λ with the following probability distribution (Marčenko & Pastur, 1967):

$$p\left(\lambda \right)=\left\{ \begin{array}{l} \frac{\sqrt{\left(\lambda_{+}-\lambda \right)\left(\lambda -\lambda_{-}\right)}}{2\pi \gamma \lambda \sigma^{2}}\;\;\;\;if\lambda_{-}-<\lambda <\lambda_{+}\\ \;\;\;\;\;\;\;\;\;\;\;\;\;\;\;\;0\;\;\;\;\;\;\;\;\;\;\;\;\;\;\;\;\;\;\;\;\;\;\;\;\;\;\;otherwise \end{array}\right.$$ (3)

where

$$\lambda_{\pm }=\sigma^{2}{\left(1\pm \sqrt{\gamma }\right)}^{2}$$ (4)

Note that this Marčenko-Pastur (MP) distribution produces non-zero probabilities only between $\lambda_{-}$ and $\lambda_{+}.$ From Eq. 4, the width of the MP distribution $\lambda_{+}-\lambda_{-}$ can be related to the noise variance as

$$\lambda_{+}-\lambda_{-}=4\sqrt{\gamma }\sigma^{2}.$$ (5)

Veraart, Novikov, et al. (2016) proposed to classify eigenvalues mostly related to noise as the maximum number of the smallest eigenvalues $\lambda_{c}$ that best fits the probability distribution in Eq. 3. In practice, this is achieved by a moment-matching method in which larger eigenvalues (carrying significant signal information) are iteratively removed until the mean of the lowest intensity eigenvalues ${\bar{\lambda }}_{c}$ is higher than a noise variance estimated from the MP distribution ($\hat{\sigma }MP^{2}$), that is:

$${\bar{\lambda }}_{c}\geq \hat{\sigma }MP^{2}$$ (6)

where the noise variance estimate $\hat{\sigma }MP^{2}$ is computed from the MP distribution bandwidth (estimated by the difference between the lower and larger eigenvalues kept in $\lambda_{c}$) according to Eq. 5:

$${\hat{\sigma }}_{MP}^{2}=\frac{\max\left(\lambda_{c}\right)-\min\left(\lambda_{c}\right)}{4\sqrt{\gamma_{c}}}$$ (7)

with $\gamma_{C}=\frac{C}{M}$ and C being the number of eigenvalues classified as being mostly related to noise. Note that MPPCA denoising does not require prior knowledge about the noise variance since $\hat{\sigma }MP^{2}$ is iteratively calculated by Eq. 7.

As an alternative to the moment-matching fitting procedure described above, the MP distribution can also be iteratively fitted to the measured PCA eigenvalue spectrum (Ding et al., 2010; Veraart, Fieremans, & Novikov, 2016). However, in this study, we show that this algorithm does not only require much longer computation times but also suffers from similar spatially correlated noise issues compared to the moment-matching algorithm (results reported in Supplementary Material).

#### 2.1.3 General PCA denoising

The denoising approach described above assumes that the eigenvalue mean ${\bar{\lambda }}_{c}$ is only larger than ${\overset{⌢}{\sigma }}_{MN}^{2}$ when all components containing relevant signal information are removed from the group of eigenvalues $\lambda_{c}$. This assumption holds for independent, uncorrelated entries in the ***X*** matrix. However, this assumption would be violated in typical MRI acquisitions where spatial correlations are introduced by reconstruction in **X**. In that case, as will also be shown below, the original MPPCA denoising considerably underperforms because it provides an incorrect classification of principal components containing mostly noise and signal information.

Therefore, instead of trying to iteratively estimate the noise variance ${\hat{\sigma }}^{2}$ from the Marčenko-Pastur distribution (an estimate that can be corrupted by spatially correlated noise), we introduce here the general PCA denoising approach, which is designed to use an a-priori noise variance estimate ${\hat{\sigma }}_{prior}^{2}$ that is obtained independently from the denoising procedure without, however, using empirical conversion factors (Manjón et al., 2013, 2015). In this study, ${\hat{\sigma }}_{prior}^{2}$ is calculated from MRI data repetitions, but the denoising approaches developed here can be adapted to other ${\hat{\sigma }}_{prior}^{2}$ estimation strategies (Aja-Fernández et al., 2015; Koay et al., 2009; Landman, Bazin, & Prince, 2009; Landman, Bazin, Smith, et al., 2009; Liu et al., 2014; Manjón et al., 2013, 2015; Samsonov & Johnson, 2004; St-Jean et al., 2020)—see, however, the considerations in section 2.1.5.

According to random matrix theory, the mean of PCA eigenvalues is only smaller than the ground-truth noise variance ${\hat{\sigma }}^{2}$ when no eigenvalue of significant signal components is improperly classified as noise (Ding et al., 2010; Manjón et al., 2015)—see also the derivations in Supplementary Material Appendix A. Given this, a way to directly use the noise variance prior is to classify eigenvalues of components containing mostly noise by selecting the highest number of smallest eigenvalues whose mean is smaller than ${\hat{\sigma }}_{prior}^{2}$, that is:

$${\bar{\lambda }}_{c}\leq {\hat{\sigma }}_{prior}^{2}$$ (8)

Note that this eigenvalue classification procedure is more general than the criteria used in conventional MPPCA since it does not rely on specific assumptions of the Marčenko-Pastur distribution (see Supplementary Material Appendix A)—which is why we refer to this denoising approach as General PCA (GPCA) denoising.

#### 2.1.4 Threshold PCA denoising

As an alternative to GPCA, the noise variance estimate ${\hat{\sigma }}_{prior}^{2}$ can also be used with an MP distribution-like concept to obtain an objective threshold for PCA component classification (Cordero-Grande et al., 2019; Moeller et al., 2021; Pai et al., 2011; Vizioli et al., 2021). Here, this is achieved by inserting ${\hat{\sigma }}_{prior}^{2}$ directly into Eq. 4. According to this equation, all noise eigenvalues of a random matrix X should be smaller than $\lambda_{+}$, and thus an eigenvalue threshold criterion for the threshold PCA denoising (TPCA) is here defined as:

$$\lambda_{t}={\left(1+\sqrt{\gamma }\right)}^{2}{\hat{\sigma }}_{prior}^{2}$$ (9)

This strategy uses the upper bound $\lambda_{+}$ of the MP distribution, but it does not require the entire distribution to follow exactly the MP distribution itself. In other words, the full shape of the eigenvalue probability spectrum is less critical than the upper bound itself. This approach avoids the deleterious effects of spatially correlated noise by avoiding the iterative calculation of two parameters ${\hat{\sigma }}_{MP}^{2}$ and ${\bar{\lambda }}_{c}$ of MPPCA (c.f. Eq. 6), and rather using the upper bound as the more important metric. Indeed, c.f. Eq. 9, where it is evident that TPCA avoids iterative computation by directly thresholding principal components using the new information from ${\hat{\sigma }}_{prior}^{2}$ which, in a way, accounts for these effects (see considerations below). It is important to note that this approach is similar to the eigenvalue thresholding approach used by Noise Reduction with Distribution Corrected (NORDIC) PCA method (Moeller et al., 2021; Vizioli et al., 2021) in which eigenvalues are classified based on an eigenvalue upper bound also computed for a noise variance estimate. However, while NORDIC uses Monte-Carlo simulations, here the analytical solution from the MP distribution is used (i.e., Eq. 9) to avoid Monte-Carlo stochastic errors—more details are given in the Discussion (section 4.3).

#### 2.1.5 Noise variance estimation

In modern MRI acquisitions, the noise level may not be uniformly constant across the entire volume (Aja-Fernández et al., 2015; Landman, Bazin, Smith, et al., 2009; Pieciak et al., 2017). Therefore, the noise variance maps in this study are computed at the voxel level. Several techniques have been proposed to calculate these maps from single MRI images (Aja-Fernández et al., 2009, 2015; Liu et al., 2014; Pieciak et al., 2017; Tabelow et al., 2015); however, to compute the effective noise variance after image reconstruction avoiding any assumption about how noise varies spatially across adjacent voxels (Constantinides et al., 1997; Landman, Bazin, & Prince, 2009; Landman, Bazin, Smith, et al., 2009; Sodickson et al., 1999), here, noise variance maps were computed from independent images acquired with identical acquisition parameters. For the diffusion MRI datasets analyzed in this study, we used multiple b-value = 0 acquisitions and calculated the signal variance across the repetitions, that is, ${\hat{\sigma }}_{prior}^{2}\left(x,y,z\right)=$$\sum_{i=1}^{r}{\left(S_{i}\left(x,y,z,b=0\right)-\bar{S}\left(x,y,z,b=0\right)\right)}^{2}/\left(r-1\right)$, where r is the number of b-value = 0 acquisitions, $S_{i}\left(x,y,z,0\right)$ is the signal acquired for a b-value = 0 acquisition at voxel position x,y,z, and $\bar{S}\left(x,y,z,b=0\right)$ is the averaged signal acquired from all b-value = 0 acquisitions at voxel position x,y,z,. Given the relatively high SNR of b-value = 0 images, this noise estimation strategy is expected to provide accurate noise variance estimates in tissue voxels (Constantinides et al., 1997; Dietrich et al., 2007; Landman, Bazin, & Prince, 2009; Landman, Bazin, Smith, et al., 2009; Sodickson et al., 1999); however, in voxels near boundaries (e.g., brain tissues near regions containing cerebrospinal fluid), these noise variance maps may be corrupted (typically overestimated) by image artifacts such as involuntary motion, cardiac pulsation, or image intensity drifts (Hansen et al., 2019; Landman, Bazin, & Prince, 2009; Landman, Bazin, Smith, et al., 2009; Tournier et al., 2011; Vos et al., 2017). These artifacts can be mitigated in TPCA and GPCA denoising by taking the median of ${\hat{\sigma }}_{prior}^{2}$ estimates from all voxels of each sliding window instance to achieve an “effective” noise variance estimate, assuming that noise is relatively uniform across each sliding window instance. Computing the final noise maps by the median of all the voxels of each sliding window instances has the advantage of improving the ${\hat{\sigma }}_{prior}^{2}$ estimation precision at a voxel level.

#### 2.1.6 Summary of the main differences between PCA denoising procedures

The denoising algorithms described above differ in the way they identify PCA components carrying (predominantly) noise and signal. The different algorithms identify the noise by removing the largest eigenvalues until:

1) MPPCA: ${\bar{\lambda }}_{c}\geq \frac{\max\left(\lambda_{c}\right)-\min\left(\lambda_{c}\right)}{4\sqrt{\gamma_{c}}}$;
2) GPCA: ${\bar{\lambda }}_{c}\leq {\hat{\sigma }}_{prior}^{2}$;
3) TPCA: $\max\left(\lambda_{c}\right)/{\left(1+\sqrt{\gamma }\right)}^{2}<{\hat{\sigma }}_{prior}^{2}$

Note that, altogether, the performance of these three different algorithms relies on four quantities which can be related to different variance estimates: 1) ${\hat{\sigma }}_{mean}^{2}={\bar{\lambda }}_{c}$ (variance estimated from mean of eigenvalues containing mostly noise); 2) ${\hat{\sigma }}_{MP}^{2}=\frac{\max\left(\lambda_{c}\right)-\min\left(\lambda_{c}\right)}{4\sqrt{\gamma_{c}}}$ (variance estimates from MP bandwidth); 3) ${\hat{\sigma }}_{prior}^{2}$ (a-priori variance estimate), and 4) ${\hat{\sigma }}_{max}^{2}=\max{\left(\lambda_{c}\right)/\left(1+\sqrt{\gamma }\right)}^{2}$ (variance estimate from the maximum eigenvalue containing mostly noise). The effects of spatially correlated noise in these quantities are here explored using simulations (vide infra).

In addition to these three algorithms, we also test the performance of MPPCA denoising (in Supplementary Material) by iteratively fitting the MP distribution to the measured PCA eigenvalue spectrum (Ding et al., 2010; Veraart, Fieremans, & Novikov, 2016)—referred to as MPPCA-slow due to its expected long processing times. Here, the MPPCA-slow algorithm is implemented according to the details described by Veraart, Fieremans, and Novikov (2016)—this implementation was also made freely available at https://github.com/RafaelNH/PCAdenoising.

### 2.2 Simulations

In this study, we first show that GPCA and TPCA denoising strategies are more robust to violations of MPPCA denoising assumptions using simulations where the ground-truth number of principal components is known a-priori. Two experiments were performed:

Experiment 1: A synthetic phantom comprising 12 × 12 voxels. Signals in this 12 × 12 grid were generated for 110 different synthetic diffusion-weighted signals, comprising 20 b-value = 0 signals and 90 diffusion weighted signals with b-values of 1, 2, and 3 ms/μm^2^ shells with 30 diffusion gradient directions in each shell. This phantom was sub-divided into 9 portions (4 × 4 voxel regions each), and diffusion-weighted signals were generated using the *Diffusion in Python* (DIPY (Garyfallidis et al., 2014; Henriques et al., 2021)) package according to a forward model containing two compartment types with different axially symmetric diffusion tensors with fixed axial and radial diffusivities (AD_1_ = 1.8 μm^2^/ms, AD_2_ = 1.5 μm^2^/ms, RD_1_ = 0 μm^2^/ms and RD_2_ = 0.5 μm^2^/ms). To produce a phantom with different signals across voxels, the relative volume fractions between compartment type 1 and 2 were set to a different value at each phantom portion (0.3, 0.35, 0.4 for the top portions, 0.6, 0.65, 0.7 for the middle portions, and 0.45, 0.50, and 0.55 for the bottom portions). For the three top and three bottom portions, signals were generated for well-aligned replicas of compartment 1 and 2 (different direction for each data portion), while for the three middle phantom portions, signals were produced for two orthogonal crossing replicas of each compartment type (i.e., total of four compartment replicas) to consider voxels containing crossing compartments.

After generating diffusion-weighted signals for each voxel, the phantom is corrupted with synthetic Gaussian noise at an SNR level of 30. All voxels were denoised simultaneously (i.e., M = 144, N = 110 for all algorithms) for all denoising approaches explored in this study (MPPCA (Veraart, Fieremans, & Novikov, 2016; Veraart, Novikov, et al., 2016), GPCA, TPCA). Since synthetic noise was uniform across voxels and uncorrupted by artifacts, the noise variance for both GPCA and TPCA was set to the voxel averaged ${\hat{\sigma }}_{prior}^{2}$ calculated as the variance of b-value = 0 signals $\langle {\hat{\sigma }}_{prior}{\left(x,y,z\right)}^{2}\rangle =$$\langle \sum_{i=1}^{r}{\left(S_{i}\left(x,y,x,0\right)-\bar{S}\left(x,y,z,0\right)\right)}^{2}/\left(r-1\right)\rangle$ —here the angle brackets represent the average across all phantom voxels. To assess the robustness of the denoising towards biases in ${\hat{\sigma }}_{prior}^{2}$, GPCA and TPCA were also run artificially changing ${\hat{\sigma }}_{prior}^{2}$ from 50% lower to 400% higher values than the true variance. In addition, the propagation of ${\hat{\sigma }}_{prior}^{2}$ errors into TPCA and GPCA was assessed in Supplementary Figure S1.

Experiment 2: We then assessed the situation in which spatially correlated noise is present. Spatially correlated noise (and signal) was generated by zero-filling three columns of the matrices at Fourier space. After corrupting the numerical phantoms with spatially correlated noise, data were denoised using the three denoising approaches. As for the previous experiment, noise variance for both GPCA and TPCA was calculated from the average of the unbiased sampled variance estimation across all image voxels.

To assess the generalizability of our results toward a) different types of spatially correlated noise, b) non-central mean distributed correlated noise, c) larger phantom sizes, and d) smaller number of diffusion MRI experiments, additional simulations were performed for spatially correlated noise due to Gaussian smoothing (Supplementary Fig. S2), for spatially correlated Rician noise (Supplementary Fig. S3), for a synthetic phantom comprising 66 × 66 voxels (Supplementary Fig. S4), and for a phantom with synthetic signals (generated for b-value = 2 ms/μm^2^) along 30 diffusion gradient direction together with 5 b-value = 0 signals (Supplementary Fig. S5). For 66 × 66 voxel phantoms, the spatially correlated noise was generated by zero-filling 16 columns of the matrices in Fourier space to maintain a similar zero-filling factor to the phantoms with 12 × 12 voxels.

Since diffusion MRI is typically used to compute parametric maps of diffusion properties, we also assessed the impact of the different denoising strategies on standard Diffusional Kurtosis imaging (DKI) (Jensen et al., 2005; Tabesh et al., 2011) metrics reconstructed from both raw and denoised signals using the DKI modules implemented in the open-source package *Diffusion in Python* (DIPY) (Henriques et al., 2021).

Note that, for all simulations above, the optimal number of signal components preserved by denoising is known a-priori to be exactly 8 since the phantom was fully constructed from 9 different portions with fixed diffusivities, directions, and volume fractions (one signal component must be removed since we subtract the mean of ***X*** before denoising). Moreover, for these simulations, the denoising performance can be directly assessed by comparing signal maps for individual diffusion gradient direction and b-values to their corresponding ground-truth maps. The ground-truth signals for Experiment 1 corresponded to the synthetic phantom signal before noise corruption, while the ground-truth signals for Experiment 2 were computed by zero-filling columns of the original noise free matrices in Fourier space.

### 2.3 Preclinical MRI experiments

All animal experiments were preapproved by the institutional and national authorities, and carried out according to European Directive 2010/63. A mouse brain (C57BL/6J) was extracted via transcardial perfusion with 4% Paraformaldehyde (PFA), immersed in 4% PFA solution for 24 h, washed in Phosphate-Buffered Saline (PBS) solution for at least 24 h, and then placed on a 10 mm NMR tube filled with Flourinert (Sigma Aldrich, Lisbon, PT), which was sealed using paraffin film.

The MRI experiments were performed on a 16.4T Bruker Aeon Ascend scanner (Bruker, Karlsruhe, Germany), interfaced with an Avance IIIHD console, and equipped with a gradient system capable of producing up to 3000 mT/m in all directions. A constant temperature of 37^o^C was maintained throughout the experiments using the probe’s variable temperature capability. Two distinct diffusion-weighted datasets were then acquired using Bruker’s standard “Diffusion Tensor Imaging EPI” sequence for the following diffusion-weighted parameters: 30 gradient directions for b-values 1, 2, and 3 ms/μm^2^ (Δ = 15 ms, δ = 1.5 ms), and 20 consecutive b-value = 0 acquisitions. Data reconstructed from EPI acquisitions are expected to be corrupted by spatially correlated noise from multiple sources (regridding, partial Fourier factor, multiple shots, including k-space sampling during gradient ramp, etc.). We modulated the amount of spatial correlations by acquiring EPI datasets with parameters optimized to mitigate noise spatial correlations, particularly avoiding k-space undersampling acquisition during EPI’s gradient ramps and without using partial Fourier. Then, a second dataset was acquired with identical resolution, number of acquisitions, and so on, but with large factors inducing spatial correlations, including k-space sampling during gradient ramps (default Bruker’s acquisition and reconstruction procedures for acquisition speed) and with a significant phase partial Fourier factor of 6/8 (note for partial Fourier acquisitions, EPI data are reconstructed with zero-padding, according to the default reconstruction procedures by Bruker’s pre-clinical reconstruction software Paravision 6.0.1). All other acquisition parameters were kept constant: TR/TE = 3000/50 ms, 9 coronal slices, Field of View = 12 × 12 mm^2^, matrix size 80 × 80, in-plane voxel resolution of 150 × 150 μm^2^, slice thickness = 0.7 mm, number of averages = 2, number of segments = 1, and double sampling acquisition. For a gold standard reference, the second dataset was also repeated for 20 averages.

Spatial drifts in the image domain were first corrected using a sub-pixel registration technique (Guizar-Sicairos et al., 2008). Due to the anisotropic voxel sizes (slice thickness > in-plane resolution), MPPCA, GPCA, and TPCA were then applied for each coronal slice separately using a 2D sliding window with 11 × 11 voxel (M = 121, N = 110). For all denoising approaches, signals from overlapping windows were combined using the overcomplete averaging procedure described in Katkovnik et al. (2009) and Manjón et al. (2013). Noise variance maps for both GPCA and TPCA were computed from all 20 consecutive b-value = 0 acquisitions—final ${\hat{\sigma }}_{prior}^{2}$ values for each sliding window iteration were taken as the median ${\hat{\sigma }}_{prior}^{2}$ value across all sliding window voxels. As for the simulations, in addition to the assessment of the denoising performance of individual diffusion-weighted signals, we also computed standard Diffusional Kurtosis Imaging (DKI) metrics from both raw and denoised data.

### 2.4 MRI experiments using a clinical scanner

Experiments were approved by the Ethical Committee of the University of Trento, and the participant signed an informed consent form. MRI data were acquired for a healthy control (male, 54 years) using a 3T MAGNETOM PRISMA scanner (Siemens Healthcare, Erlangen, Germany) equipped with a 64-channel head-neck RF receive coil. Diffusion MRI data were acquired using a monopolar single diffusion encoding EPI PGSE (Feinberg et al., 2010; Moeller et al., 2010; Xu et al., 2013) along 30 diffusion gradient directions for 5 non-zero b-values = 1, 2, 3, 4.5, and 6 ms/μm^2^ (Δ = 39.1 ms, δ = 26.3 ms) and 17 interspersed b-value = 0 acquisitions. Other acquisition parameters were the following: TR/TE = 4000/80 ms, 63 axial slices, Field of View = 220 × 220 mm^2^, matrix size 110 × 110, isotropic resolution of 2 mm, 6/8 phase partial Fourier, parallel imaging with GRAPPA 2, and simultaneous multi-slice factor 3. All diffusion MRI data were reconstructed using zero-padding, which is the default procedure for data acquired with partial Fourier above 70%.

MPPCA, GPCA, and TPCA denoising were applied with a 3D sliding window of 9 × 9 × 3 voxels to perform PCA denoising on a number of voxels (*M* = 242) similar to the number of diffusion experiments (*N* = 167), while maintaining matrices with *M > N* to achieve higher denoising SNR gains. Higher sliding windows were not considered to minimize the effects of spatially varying noise level across the voxels of different sliding window instances (c.f. discussion in section 4.4). To minimize the effects of image artifacts on interspersed b-value = 0 acquisitions, initial ${\hat{\sigma }}_{prior}^{2}$ estimates for both GPCA and TPCA were computed from the first five b-value = 0 images, and final ${\hat{\sigma }}_{prior}^{2}$ estimates were taken as the median ${\hat{\sigma }}_{prior}^{2}$ value for each sliding window.

As for the pre-clinical data, denoising performance was assessed on individual diffusion-weighted signals and on diffusion parametric maps. Since DKI fails to represent the diffusion signal decays at high b-values (Chuhutin et al., 2017; Jensen & Helpern, 2010), its maps are reconstructed from the raw/denoised signal for b-value ≤3 ms/μm^2^. Additionally, to inspect the preservation of the diffusion MRI angular information at high b-value data, the data for b-values = 4.5 and 6 ms/μm^2^ and respective interspersed b-value = 0 acquisitions were subsampled and used to reconstruct Q-ball diffusion orientation distribution functions (dODFs) using the constant solid angle procedure (Aganj et al., 2010) also implemented in DIPY.

## 3 Results

### 3.1 Numerical simulations

#### 3.1.1 Scenario A: Spatially uncorrelated noise

**Fig. 1.**
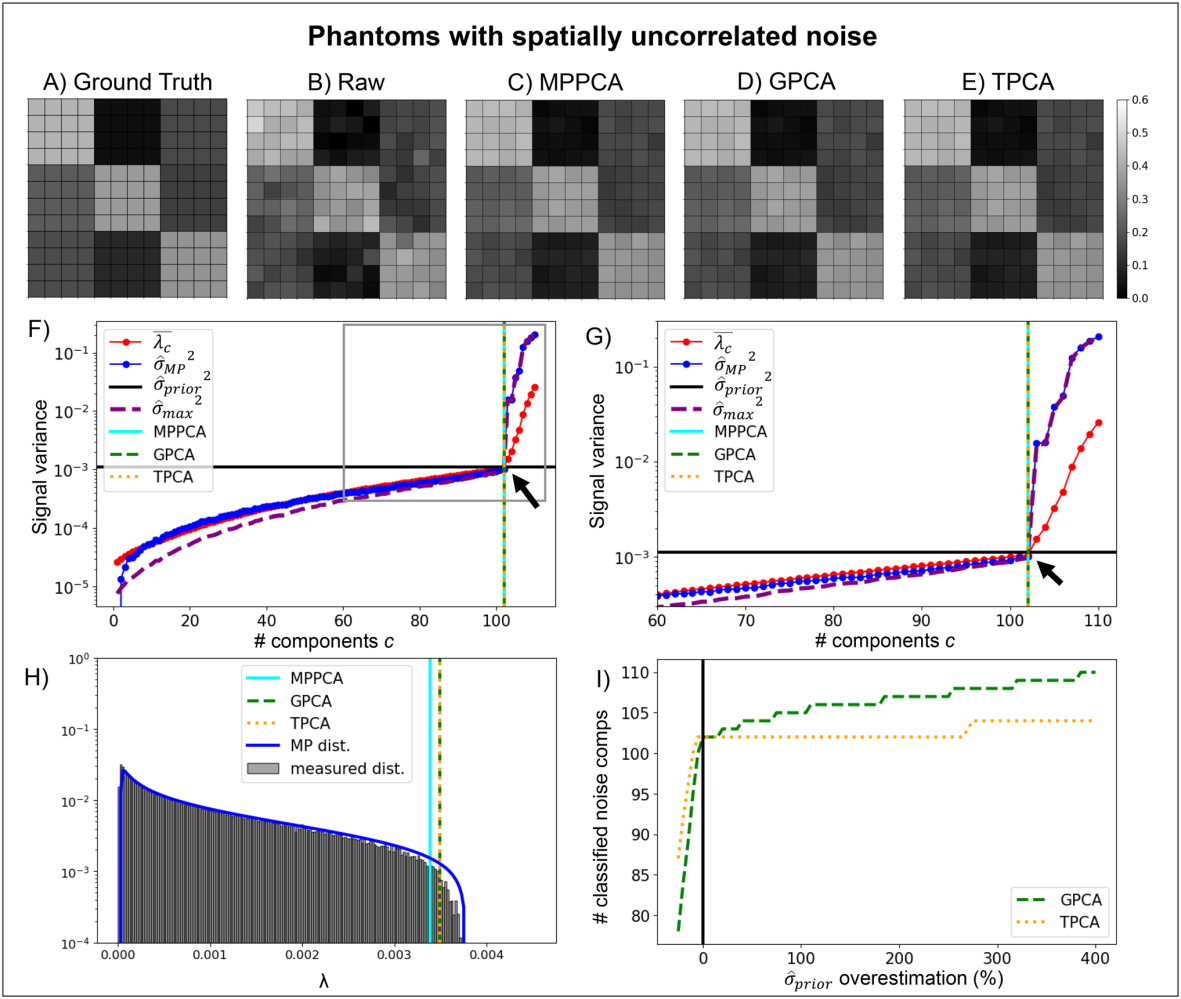
Simulations of denoising performance in a phantom with uncorrelated noise. Representative ground truth, noise free **(A)**, and noise corrupted signals **(B)**, for the first diffusion gradient direction of the highest diffusion gradient intensity are shown in panels respectively, while denoised signals for the MPPCA **(C)**, GPCA **(D)**, and TPCA **(E)** denoising algorithms. **(F)** Parameters assessed by the denoising algorithms plotted as a function of the number of lower eigenvalues potentially considered as noise. Thresholds for the MPPCA, GPCA, and TPCA are plotted by the cyan solid, green dashed, and orange vertical lines respectively (black arrow points to the ground-truth number of signal components, i.e., 102). **(G)** Zoomed plot of the parameters assessed by the denoising algorithms. **(H)** Reconstructed eigenvalue spectrum for 1000 trials and respective theoretical MP distribution for identical eigenvalue variances are shown in panel—the median thresholds for the MPPCA, GPCA, and TPCA computed as the threshold median across the 1000 repetitions are plotted by the cyan solid, green dashed, and orange vertical lines respectively. **(I)** The number of classified noise components for both GPCA and TPCA as a function of the percentage overestimation of noise standard deviation. All algorithms produced identical denoising performances when noise is spatially uncorrelated.

To provide ground truth and validate that all methods perform similarly under ideal conditions, simulations for a phantom with spatially uncorrelated noise are shown in Figure 1. Ground truth, raw noisy, and denoised diffusion-weighted signals for the first diffusion gradient direction of the highest diffusion gradient magnitude simulated (b = 3 ms/μm^2^) are shown in upper panels (Fig. 1A-E). In Figure 1F (and Fig. 1G for zoomed plotted), the quantities ${\bar{\lambda }}_{c}$, ${\hat{\sigma }}_{MP}^{2}$,${\hat{\sigma }}_{prior}^{2}$, and ${\hat{\sigma }}_{max}^{2}=\max{\left(\lambda_{c}\right)/\left(1+\sqrt{\vartheta }\right)}^{2}$ evaluated by the denoising algorithms to classify PCA eigenvalues are plotted. In Figure 1E and Figure 1G, the number of noise components classified by the denoising algorithms are marked by the vertical lines (cyan, green, and orange vertical lines for MPPCA, GPCA, and TPCA, respectively). All algorithms successfully classified the 102 components containing mostly noise and the 8 signal components when noise was spatially uncorrelated as evident by the coincidence of the vertical lines (c.f. black arrows in Fig. 1A3). Figure 1H shows the eigenvalue spectrum reconstructed by repeating the simulations 1000 times and the theoretical MP distribution with equal eigenvalue variance (plotted in log scale for better visalization of the lower probabilities for higher eigenvalues). As expected, the reconstructed spectrum matches the theoretical MP distribution when noise is spatially uncorrelated. For all PCA denoising algorithms, the threshold median across the 1000 simulation repetitions approaches the distribution upper bound (vertical lines in Fig. 1H). Since all algorithms classified identical number of signal components, their performances are qualitatively indistinguishable from each other (c.f. Fig. 1A-E). To test how these methods perform in case of ${\hat{\sigma }}_{prior}$ overestimation, the number of components classified as mostly containing noise are plotted in Figure 1I for different ${\hat{\sigma }}_{prior}$ underestimation and overestimation factors. TPCA is more robust to ${\hat{\sigma }}_{prior}$ misestimation than GPCA.

#### 3.1.2 Scenario B: Spatially correlated noise

**Fig. 2.**
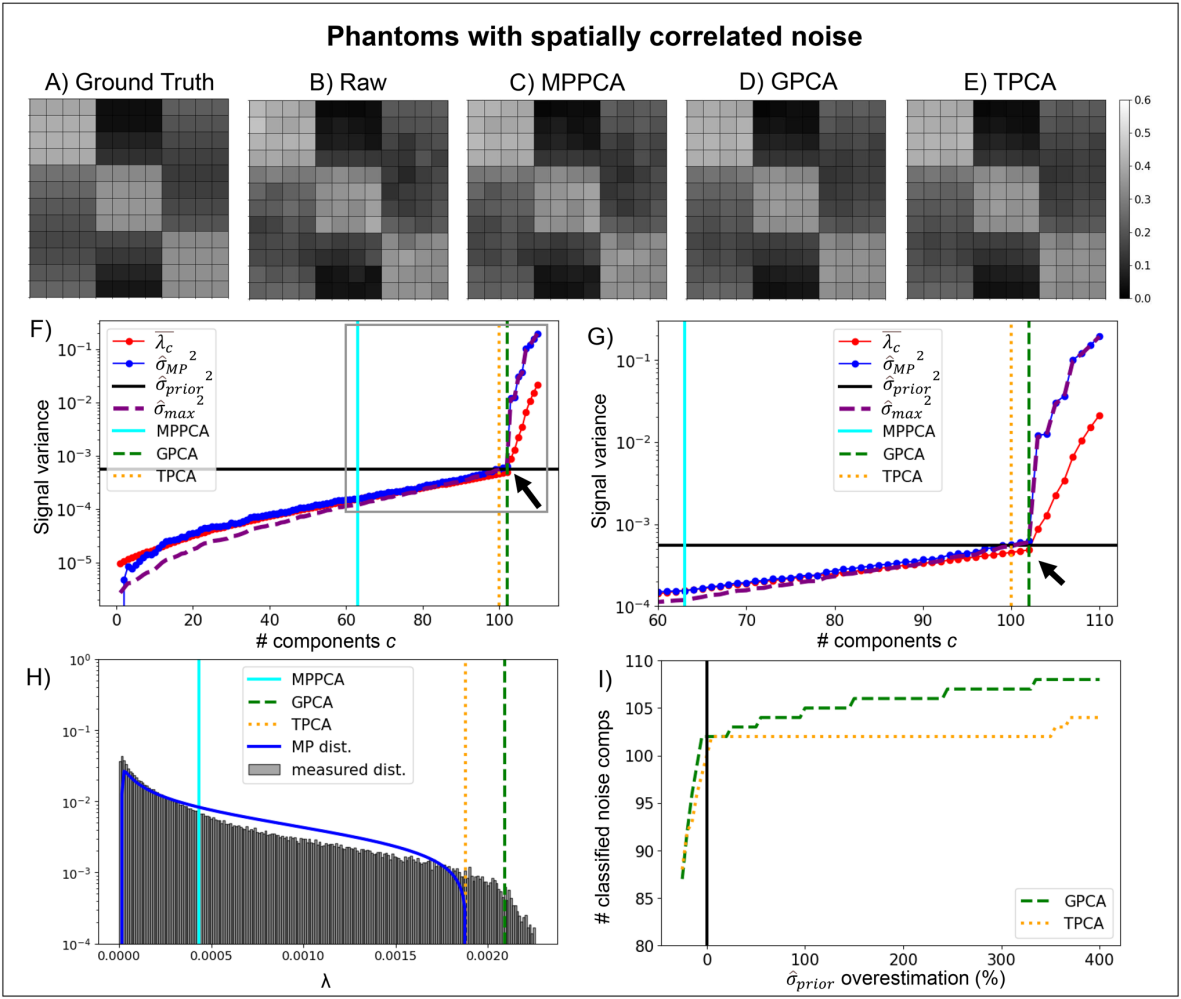
Simulations of denoising performance in a phantom with correlated noise. Correlations were induced by zero filling in k-space. Representative ground-truth noise free **(A)** and noise corrupted **(B)** signals for the first diffusion gradient direction of the highest diffusion gradient intensity alongside denoised signals for the MPPCA **(C)**, GPCA **(D)**, and TPCA **(E)**. **(F)** Parameters assessed by the denoising algorithms are plotted as a function of the number of lower eigenvalues potentially considered as noise. Thresholds for the MPPCA, GPCA, and TPCA are plotted by the cyan solid, green dashed, and orange vertical lines respectively (black arrow points to the ground-truth number of signal components, i.e., 102). **(G)** Zoomed plot of the parameters assessed by the denoising algorithms. **(H)** Reconstructed eigenvalue spectrum for 1000 instantiations and respective theoretical MP distribution for identical eigenvalue variances. The median thresholds for the MPPCA, GPCA, and TPCA computed as the threshold median across the 1000 repetitions are plotted by the cyan solid, green dashed, and orange vertical lines respectively. **(I)** The number of classified noise components for both GPCA and TPCA as a function of the percentage overestimation of noise standard deviation. Thus, GPCA and TPCA denoising algorithms outperform MPPCA denoising when noise is spatially correlated.

Next, we investigate the phantom with spatial correlations (Fig. 2) by zero-filling 1/4 data columns in Fourier space, which interpolates the data in image domain and creates strong spatial correlations. Equivalent results with another means of creating spatial correlations are shown in Supplementary Figure S2, and Supplementary Figure S3, where correlated noise is generated by Gaussian smoothing and zero-filling correlated Rician noise, respectively. Ground truth, raw noisy, and denoised diffusion-weighted signals for a selected diffusion gradient direction of the highest diffusion gradient magnitude simulated (b = 3 ms/μm^2^) are presented in Figure 2A-E. The MPPCA denoising classification criterion $\left({\bar{\lambda }}_{c}\geq {\hat{\sigma }}_{MP}^{2}\right)$ identified only a small number of PCA eigenvalues as mostly carrying noise (solid cyan line in Fig. 2F). That is, the classification procedure underestimated the number of noise components, thereby negatively impacting denoising performance.

In contrast to the MPPCA, GPCA denoising correctly classified all 102 components containing mostly noise and 8 signal components (dashed orange line in Fig. 2F and Fig. 2G) based on the criterion ${\bar{\lambda }}_{c}\leq {\hat{\sigma }}_{prior}^{2}$. TPCA misclassified 2 ground-truth noise components as being significant signal components (${\hat{\sigma }}_{max}^{2}=\;<{\hat{\sigma }}_{prior}^{2}$, dashed green line in Fig. 2F and Fig. 2G), but still exhibited good denoising performance without removing signal components.

Figure 2H shows that the bandwidth of the eigenvalue spectrum for eigenvalues of spatially correlated noise is increased relative to the theoretical MP distribution with equal eigenvalue variance. Qualitatively, while the denoised signals from the MPPCA (Fig. 2C) were nearly identical to the raw non-denoised signals (Fig. 2B), the denoised signals from GPCA (Fig. 2D) and TPCA (Fig. 2E) corresponded much better to their respective ground truth maps (Fig. 2A). As for the previous scenario, TPCA is more robust to ${\hat{\sigma }}_{prior}$ misestimation than GPCA, even for spatially correlated noise (Fig. 2I). The performance of GPCA and TPCA was shown to be stable even for larger phantom with spatially correlated noise (Supplementary Fig. S4) and for phantoms with smaller number of diffusion MRI experiments (Supplementary Fig. S5).

#### 3.1.3 Diffusion parametric maps

**Fig. 3.**
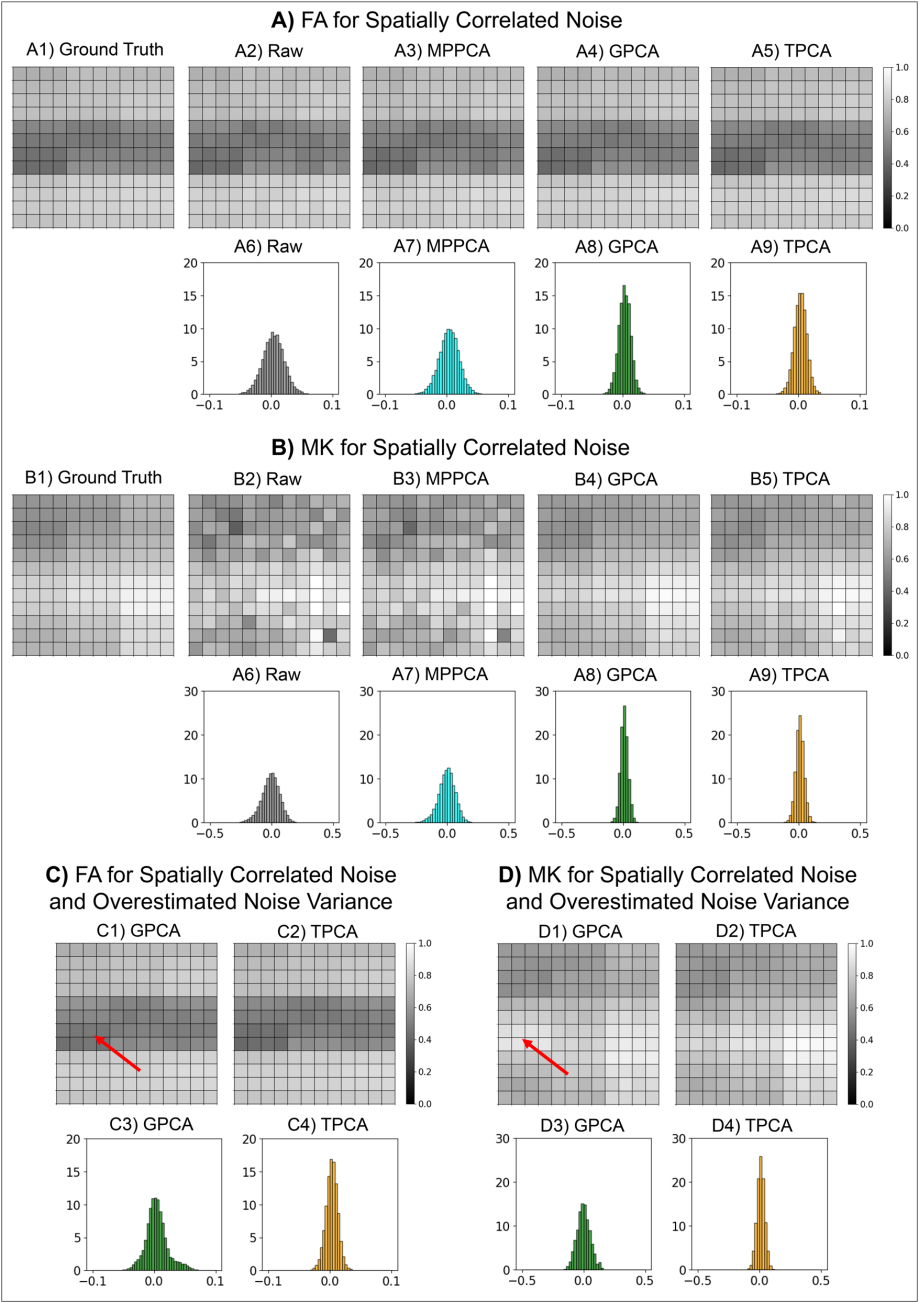
Simulated denoising performance in FA and MK estimates. In panels **(A)** and **(B)**, upper panels show the maps computed from ground truth (A1, B1), noise corrupted (A2, B2), MPPCA denoised (A3, B3), GPCA denoised (A4, B4), and TPCA denoised (A5, B5) signals, while lower panels show the FA/MK residual histograms computed by repeating 100 noise instances (corresponding to a total of 14400 = 144 voxels per phantom × 100 noisy phantom instances) for raw and all denoising strategies. In panels **(C)** and **(D)**, upper panels show the maps computed from GPCA (C1, D1) and TPCA (C2, D2) denoised signals and respective residuals histograms (C3, D3, C4, D4) when overestimated noise variance (200% of its original value) is used. Red arrow in panels (C1, D1) indicates regions with biased FA/MK estimates. This figure shows that GPCA provides optimal denoising performance in this case where the noise variance is accurately estimated, while TPCA provides the optimal performance in this case where the noise variance is overestimated.

Next, we assess the impact of different denoising strategies on extracted DKI parameters. For the sake of simplicity, only the results for FA and MK estimates extracted from the phantoms corrupted by spatially correlated noise are presented in Figure 3A-B. As for the synthetic diffusion-weighted signal, FA and MK maps from the denoised data for both GPCA and TPCA (Fig. 3A4-5 and Fig. 3B4-5) show a better resemblance to the ground-truth maps (Fig. 3A1 and Fig. 3B1) than the FA and MK maps obtained from the raw and MPPCA denoised data (Fig. 3A2-3 and Fig. 3B2-3). For a quantitative characterization of denoising performance of DKI estimates, simulations were repeated over 100 noise instances, and histograms of errors were computed by subtracting FA and MK estimates from their ground-truth values (Fig. 3A6-9 and Fig. 3A6-9). As expected, smaller error amplitudes are seen for FA and MK estimates with the GPCA and TPCA denoised data.

The above analysis was repeated for FA and MK estimates extracted from GPCA and TPCA denoised data when ${\hat{\sigma }}_{prior}$ is overestimated by 200% of its original values (Fig. 3C-D). Maps obtained for GPCA denoised data show voxels with biased FA and MK (red arrows in Fig. 3C1 and Fig. 3D1). This observation is confirmed by the larger residual histogram widths in Figure 3C3 and Figure 3D3. As for Figure 1 and Figure 2, TPCA is seen to be robust to ${\hat{\sigma }}_{prior}$ overestimations (Fig. 3C2, Fig. 3C4, Fig. 3D2, and Fig. 3D4). Root-mean-squared errors for FA/MK and other DKI estimates are reported in Supplementary Table S1. Angular errors of compartment direction estimates extracted from Q-ball orientation distribution function reconstructions using the highest b-value signals (together with b-value = 0 signal repetitions) are shown in Supplementary Figure S6. As for the FA and MK quantities, smaller angular errors are observed for data denoised by GPCA and TPCA when the noise variance is accurately estimated; however, larger angular errors are present for data denoised by GPCA when the noise variance is overestimated.

### 3.2 Preclinical MRI experiments

#### 3.2.1 Data acquired with small spatial correlations

**Fig. 4.**
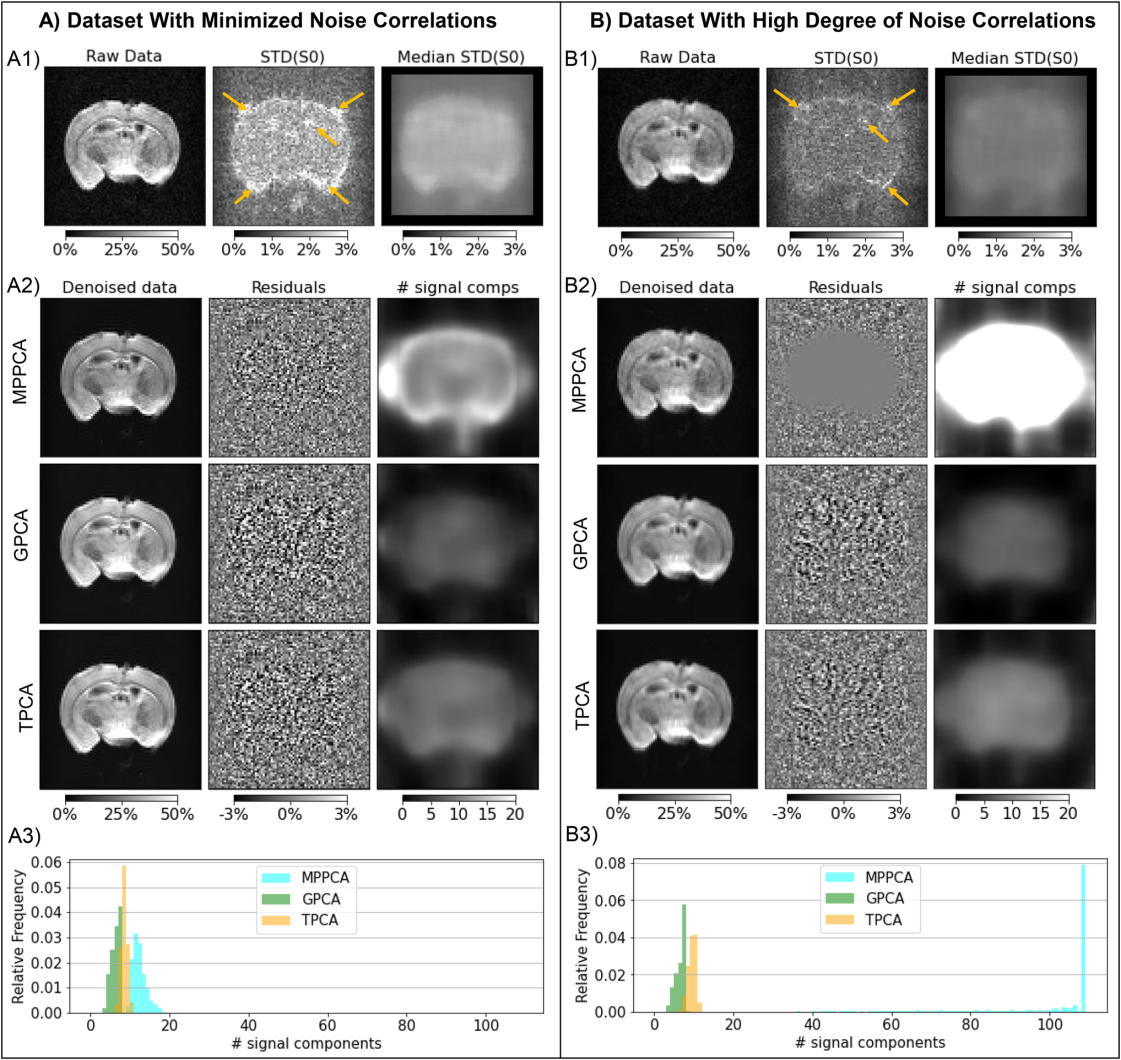
Denoising performance in preclinical datasets. **(A)** Uncorrelated noise scenario and **(B)** acquisition with much higher noise correlations. From left to right, the upper panels (A1/B1) show a representative diffusion MRI slice for a selected gradient direction acquired with b-value = 3 ms/µm^2^, the initial noise standard deviation (STD) maps computed as the standard deviation of the signals across the data for different b-value = 0 acquisitions, and finally the noise STD maps computed after selecting the median values across the voxels of the PCA sliding windows (yellow arrows point regions of high STD noise estimates). From left to right, the lower panels (A2/B2) show the denoised data for the selected diffusion MRI image, the denoising residuals computed as the difference between denoised and raw data, and the number of PCA signal components preserved by each denoising algorithm (upper to lower panels shows the results for MPPCA, GPCA, and TPCA respectively). (A3/B3) shows the relative frequencies of the number of PCA signal components preserved by each denoising algorithm across all brain voxels. The map intensities for raw and denoised data as well as noise STD estimates and denoising residuals are displayed in reference to the mean b = 0 signals across all brain voxels (a value of 100% corresponds to values equal to that reference value). All denoising algorithms have similar performances on data with minimal spatially correlated noise **(A)**; however, MPPCA fails to denoise data highly corrupted by spatially correlated noise while both GPCA and TPCA maintain their performance **(B)**.

Results for the diffusion-weighted data acquired with parameters adjusted to minimize noise spatial correlations are presented in Figure 4A. From left to right, the upper panel (Fig. 4A1) shows a raw representative image acquired with a b-value of 3 ms/µm^2^, the initial noise standard deviation (${\hat{\sigma }}_{prior}$) map before performing the median filtering across the voxels of the PCA denoising sliding windows, and the final noise standard deviation (${\hat{\sigma }}_{prior}$) map after selecting the median value of each sliding window, which was used in the subsequent denoising procedures. The initial noise standard deviation map (middle image of Fig. 4A1) was spatially uniform across the brain, except for higher values near brain edges likely due to small image intensity drifts and residual spatial drifts (yellow arrows in Fig. 4A1). These higher noise variance values were successfully attenuated after selecting the median values across sliding windows (right image of Fig. 4A1).

The lower panel (Fig. 4A2) shows the corresponding denoised diffusion-weighted image, the denoising residuals computed as the subtraction between the denoised and raw representative image, and the number of classified signal components for all three denoising algorithms tested—MPPCA, GPCA, and TPCA from top to bottom. For this dataset with a few spatial correlations, all denoising algorithms performed similarly. For instance, all strategies uniformly classified around 10 PCA signal components across the brain (Fig. 4A2 and Fig. 4A3), except in regions near the brain ventricles and boundaries in which the MPPCA classified a larger number of signal components (Fig. 4A2). Still, the residual maps are clearly similar and show no structure (Fig. 4A2).

#### 3.2.2 Dataset acquired with large noise correlations

Figure 4B shows the results for diffusion-weighted data acquired with factors inducing significant spatial correlations, including partial Fourier and signal acquisition during EPI’s gradient ramp (no ramp compensation). These are much more commonly used settings than those shown in Figure 4A. Representative images are shown in Figure 4B1 left, while noise standard deviation maps (${\hat{\sigma }}_{prior}$) before and after selecting the median values across the voxels of PCA denoising sliding windows are shown in the middle and right images of Figure 4B1.

In this type of data with spatial noise correlations, the MPPCA denoising failed: residuals are close to zero across all brain voxels and more than 40 components were classified as significant signal components (upper middle and right images of Fig. 4B2 and Fig. 4B3). We then tested whether GPCA and TPCA could outperform the MPPCA. Indeed, a uniform denoising performance across the brain and background regions was clearly observed for these two algorithms (lower rows of panels in Fig. 4B2). As the previous dataset in Figure 4A, GPCA and TPCA preserve around 10 PCA components across the brain (Fig. 4B2 right panels and Fig. 4B3).

#### 3.2.3 DKI parametric maps

**Fig. 5.**
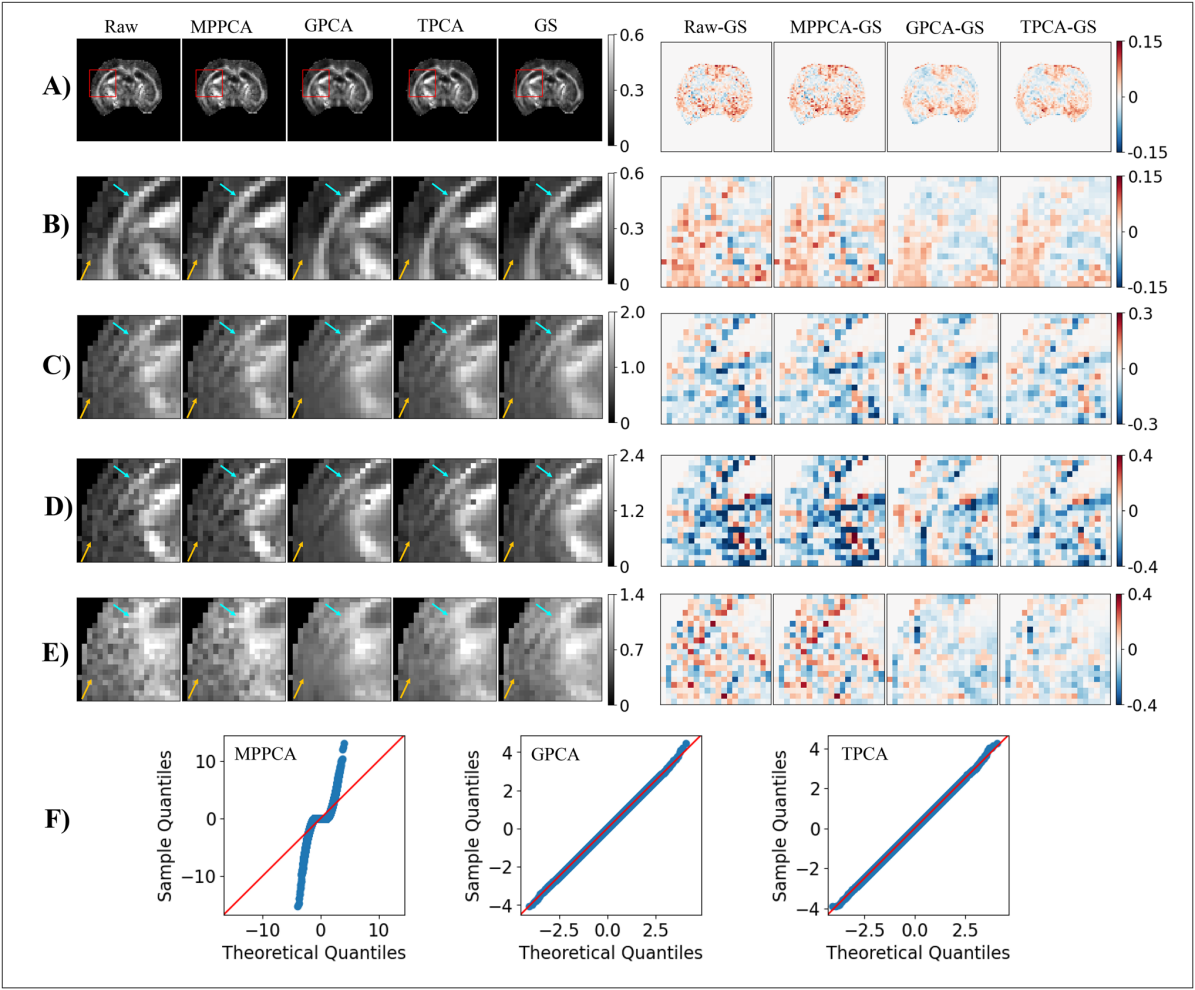
DKI maps for the pre-clinical dataset highly corrupted by spatially correlated noise and QQ-plots of the diffusion-weighted signal denoising residuals: **(A)** Fractional Anisotropy for an entire representative axial slice; **(B)** Fractional Anisotropy for the zoomed area marked by the red box; **(C)** Mean Kurtosis for the zoomed area; **(D)** Radial Kurtosis for the zoomed area; **(E)** Axial Kurtosis for the zoomed area; and **(F)** QQ-plots of the denoising residuals. Left panels show the DKI maps computed from the raw data, data denoised by MPPCA, GPCA, TPCA, and the raw data acquired for 10 times more averages (gold standard reference), while right panels show the difference between maps from raw/denoised data and their respective gold standard references. Orange arrows point to areas where noise estimate fluctuations are visually reduced, while blue arrows point to boundary areas between gray and white matter. QQ-plots of the denoising residuals for the selected zoomed region are show panel **(F)**. Note that, even when noise is highly spatially correlated, reconstruction DKI is improved on data denoised by the GPCA and TPCA procedures.

Figure 5 shows the parametric maps for four DKI maps computed from the data acquired with factors inducing significant spatial correlations (DKI maps for the dataset acquired with less spatial correlations are presented in Supplementary Fig. S7). Fractional Anisotropy (FA) results are shown for an entire representative data slice (panel A) and for the zoomed area marked by the red box (panel B), while maps for Mean Kurtosis (MK), Radial Kurtosis (RK), and Axial Kurtosis (AK) are only shown for the zoomed area for the sake of simplicity (panels C-E). For the left panels, DKI maps were separately computed from the raw data, data denoised by MPPCA, GPCA, TPCA, and the raw data acquired for 10 times more averages (gold standard reference). The right panels on the right of Figure 5 show the difference between maps from raw/denoised data and their respective gold standard references. Since MPPCA failed to effectively suppress noise for this dataset (c.f. Fig. 4B), DKI maps appear very similar between the non-denoised and MPPCA denoised data. By contrast, DKI maps computed from data denoised by the GPCA and TPCA algorithms evidence better maps, decreases in spurious fluctuations (orange arrows in Fig. 5B-E), and preservation of contrast between white and gray matter tissues (cyan arrows in Fig. 5B-E). Better performance of GPCA and TPCA is also supported by the lower differences observed between their respective DKI maps and gold standard results. Qualitatively, GPCA and TPCA evidenced very similar denoising performances.

To better quantify these effects, QQ-plots (Wilk & Gnanadesikan, 1968) of the denoising diffusion-weighted residuals for the selected zoomed region of interest are shown in Figure 5F. For both GPCA and TPCA, the sampled residual quantiles closely matched the theoretical quantiles of a Gaussian distribution, as evident from the linear nature of the plot. By contrast, substantial deviations between MPPCA sampled residual quantile and the theoretical Gaussian distribution reference reflect the poor denoising performance of MPPCA for the pre-clinical dataset acquired with no concerns on spatially correlated noise.

### 3.3 MRI experiments using a clinical scanner

#### 3.3.1 Diffusion-weighted images

**Fig. 6.**
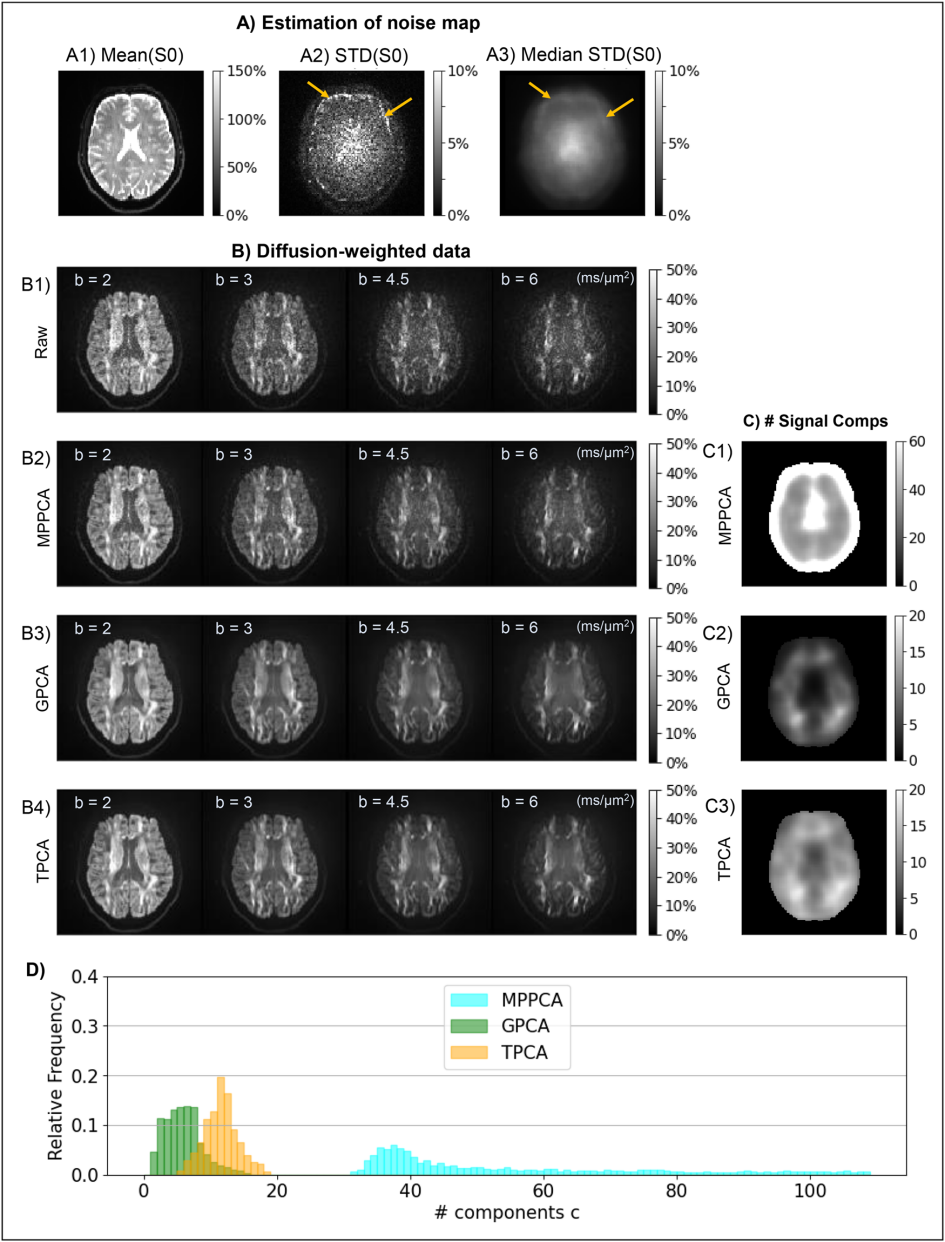
Denoising performance in diffusion-weighted data acquired using a clinical scanner. **(A)** Images related to the noise prior estimation: (A1) representative slice of the averaged signals of the five first b-value = 0 acquisitions; (A2) initial noise standard deviation (STD) maps computed as the standard deviation of the 5 first b-value = 0 acquisitions; and (A3) the final noise STD maps computed after selecting the median values across the voxels of the PCA sliding windows—orange arrows point regions of high STD noise estimates. **(B)** Representative slice of the diffusion-weighted data for a gradient direction near **v** = [1, 0. 0] and for b-value = 2, 3, 4.5, and 6 ms/µm^2^ (from left to right) before denoising (B1) and after denoising using the MPPCA (B2), GPCA (B3), and TPCA (B4) procedures. **(C)** Number of PCA signal components preserved by the four denoising algorithms: MPPCA (C1); GPCA (C2); and TPCA (C3). **(D)** Relative frequencies of the number of PCA signal components preserved by the four denoising algorithms. The map intensities for raw and denoised data as well as noise STD estimates and denoising residuals are displayed in reference to the mean b-value = 0 signals across all brain voxels (a value of 100% corresponds to values equal to that reference value). GPCA and TPCA have superior denoising performance over MPPCA in a typical high b-value diffusion-weighted dataset.

Figure 6 shows results from a healthy volunteer. Figure 6A shows the mean signal of the 5 repeating b = 0 acquisitions (Fig. 6A1), the initial ${\hat{\sigma }}_{prior}$ map computed from these b = 0 acquisitions (Fig. 6A2), and the final “effective ${\hat{\sigma }}_{prior}$” map after selecting the median values from PCA denoising sliding windows (Fig. 6A3). Higher initial ${\hat{\sigma }}_{prior}$ estimates are observed in regions near the ventricles and cortical boundaries (e.g., orange arrows in Fig. 6A2), likely an effect of signal fluctuations in these regions due to pulsation artifacts (Tournier et al., 2011). These higher noise variance estimates are suppressed but not completely removed upon median filtered ${\hat{\sigma }}_{prior}$ maps (e.g., orange arrows in Fig. 6A3).

Representative images of the raw and denoised diffusion-weighted data for the 4 higher non-zero b-values (b-value = 2, 3, 4.5, and 6 ms/µm^2^) are shown in Figure 6B. The number of preserved signal component maps for all three denoising procedures is shown in Figure 6C, while histograms are shown in Figure 6D. For this dataset, GPCA and TPCA improved denoising performance over MPPCA (Fig. 6B3/Fig. 6B4 vs. Fig. 6B2). Specifically, while MPPCA classified over 30 of the 167 PCA components as significant signal components (Fig. 6C1 and Fig. 6D), GPCA and TPCA classified less than 20 signal components across all brain regions (Fig. 6C2 and Fig. 6C3). TPCA tended to classify slightly more signal components than GPCA (Fig. 6C2-3 and Fig. 6D). Denoising residuals for each procedure are shown in Supplementary Fig. S8. GPCA produced higher amplitude residuals. Qualitatively, no structural information is observed in these residual maps.

#### 3.3.2 Diffusion parametric maps

**Fig. 7.**
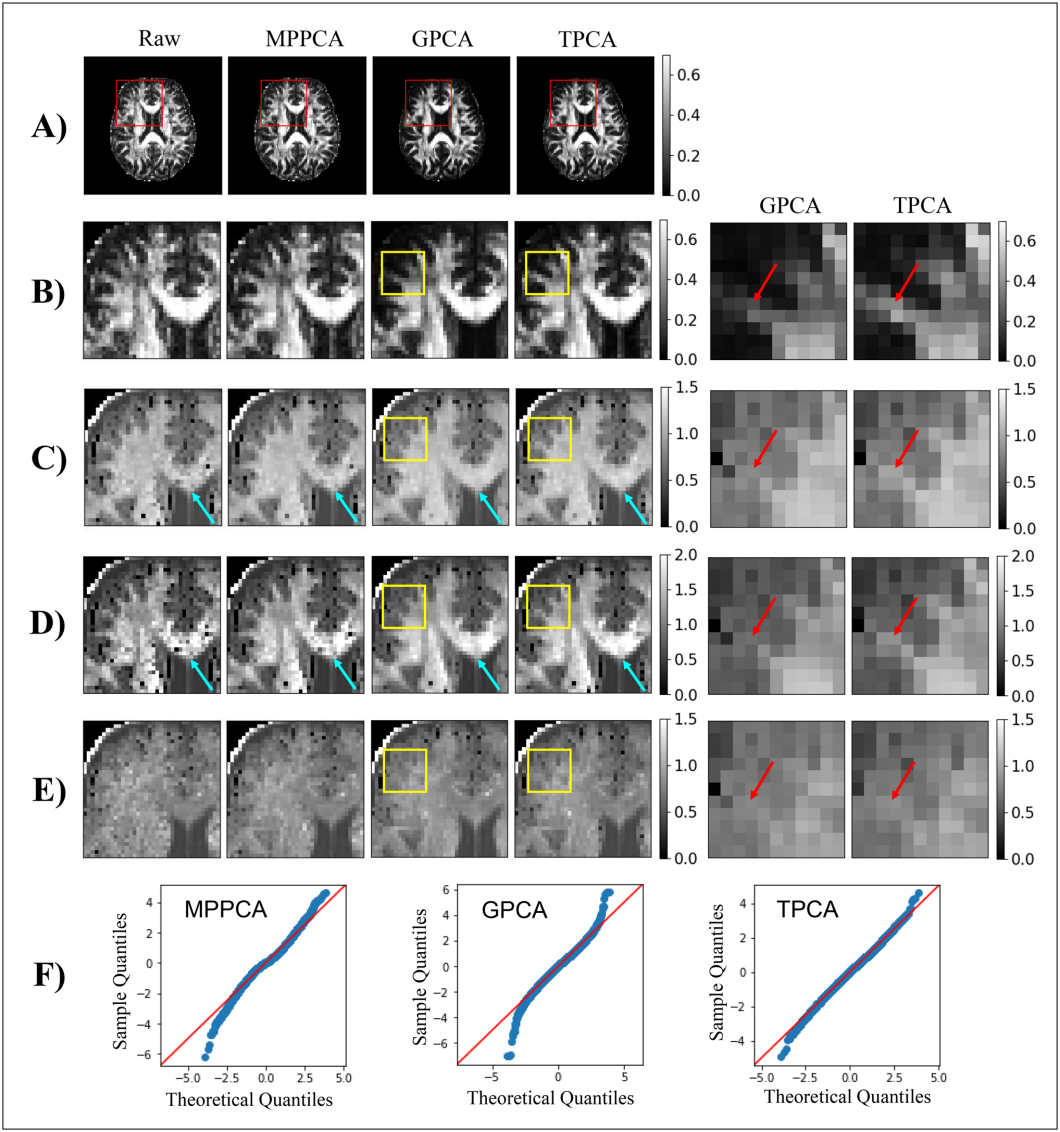
DKI maps for the diffusion-weighted human data computed for all data with b-value ≤3 ms/µm^2^ and QQ-plots of the diffusion-weighted signal denoising residuals. **(A)** Fractional Anisotropy for an entire representative slice; **(B)** Zoomed Fractional Anisotropy maps; **(C)** Zoomed Mean Kurtosis maps; **(D)** Zoomed Radial Kurtosis maps; **(E)** Zoomed Axial Kurtosis maps; and **(F)** QQ-plots of the denoising residuals in the selected yellow region of interest. In the left of panels **(B-E)**, maps extracted from raw, MPPCA, GPCA, and TPA are displayed for the zoomed area marked by the red box in panel **(A)**. Likewise, in the right of panels **(B-E)**, maps extracted from GPCA and TPA are displayed for the zoomed area marked by the yellow boxes in left images of panels **(B-E)**. GPCA and TPCA enhance the quality of DKI reconstruction for the human data (e.g., regions pointed by the cyan arrows in panels **B**, **C**, **D**, and **E**). However, while GPCA induces decreased FA, MK, RK, and AK estimates for regions near brain edges, TPCA provides an optimal compromise between noise suppression and signal preservation at these problematic regions.

Figure 7 shows sample DKI parametric maps computed from raw and denoised human brain data. As for the pre-clinical data presented above, better noise suppression was observed in diffusional kurtosis quantities (i.e., MK, RK, AK) for data denoised by GPCA and TPCA procedures compared with the MPPCA counterpart. Note, for example, the reduction of implausible negative kurtosis values in MK and RK maps for GPCA and TPCA denoised data (e.g., cyan arrows in the left panels of Fig. 7C and Fig. 7D). After a closer inspection of DKI estimates in regions near brain edges (regions of interest marked by the yellow boxes), decreases in FA, MK, RK, and AK were observed for the data denoised by the GPCA denoising strategy (red arrows in the right panels of Fig. 7B-E), suggesting that some true signal was also removed. For both MPPCA and GPCA denoising, QQ-plots of the denoising residuals of all diffusion-weighted signals in this region of interest show substantial deviations between the sampled residual quantiles and the theoretical Gaussian distribution reference (Fig. 7F). While sampled residual quantile deviations for MPPCA reflect poor removal of components mostly containing noise, deviations for GPCA indicate loss of signal information in the selected region of interest. The closer match between sampled residual quantiles and the theoretical Gaussian quantiles for TPCA suggests that this algorithm provides a better compromise between noise suppression and signal preservation.

**Fig. 8.**
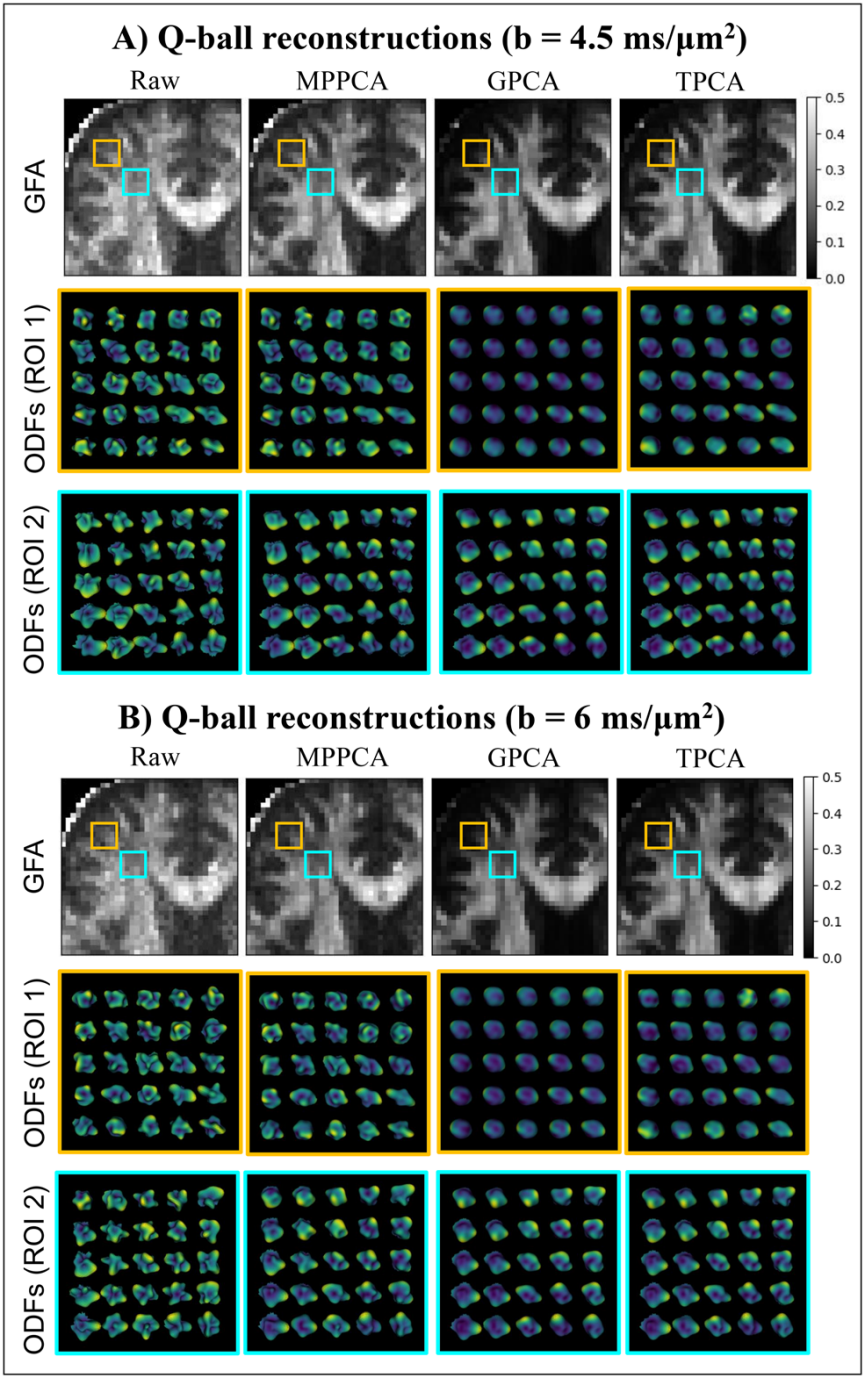
Q-ball GFA maps and ODF reconstruction for the human diffusion-weighted data independently computed for the two higher b-values: **(A)** b-value = 4.5 ms/µm^2^; and **(B)** b-value = 6 ms/µm^2^. For the raw and denoised data (different image columns), each panel shows the GFA maps for the analogous zoomed region manually defined by the red box in Figure 7A (upper row of images), and the ODFs reconstructed from zoomed orange and cyan regions defined the GFA maps (middle and lower rows of images). GPCA and TPCA reduce implausible lobes in Q-ball ODF reconstructions. ODF reconstructions from TPCA, however, produce sharper profiles in regions near brain edges (ROI 1).

To further examine the potential utility of these denoising schemes, general fractional anisotropies (GFA) maps and diffusion orientation distribution functions (ODF) are separately computed for the two higher b-values of the from raw and denoised human brain data using constant solid angle Q-ball reconstruction (Fig. 8). Consistent with the DKI results presented above, MPPCA denoised the data quite poorly, while GPCA and TPCA removed noise efficiently, but with GPCA apparently removing some signal components (c.f. regions marked by the orange boxes in the upper panels of Fig. 8A-B).

To visualize ODF reconstruction differences, the ODFs reconstructed from different version of raw and denoised data are displayed for the two regions of interest and are manually defined in upper panels of Figure 8A-B: 1) a region comprising voxels of lateral corpus callosum fiber projections where the previous FA, MK, AK, and aK for GPCA denoised data showed decreased values (ROI 1): and 2) a region comprising voxels where white matter fibers are expected to cross (ROI 2). The ODFs reconstructed from the raw and MPPCA denoised data show implausible multiple lobes for both ROIs (two last rows of images in Fig. 8A-B). Consequently, these reconstructions poorly characterized the differences expected between ODFs in the lateral single fiber corpus callosum projections (ROI1) and in the crossing fiber region (ROI2). The ODF reconstructed from the GPCA and TPCA denoised data shows the expected single and multiple lobes for ROI 1 and ROI 2 respectively; ODFs reconstructed from TPCA show, however, shaper profiles, indicating that this denoising procedure better conserves diffusion angular information. GPCA and TPCA exhibited higher reproducibility of ODF profiles across the data from the two independent b-values 4.5 and 6 ms/μm^2^. Note that ODF profiles between b-values 4.5 and 6 ms/μm^2^ data are only consistent when denoised by GPCA and TPCA algorithms (Fig. 8A vs. Fig. 8B), but not with MPPCA.

## 4 Discussion

Denoising has become a critical component of data analysis in quantitative neuroimaging (Ades-Aron et al., 2018; Adhikari et al., 2019; Diao et al., 2021; K. Kay, 2022; K. N. Kay et al., 2013; Tax et al., 2022). The existing approaches vary from subjective (PCA thresholding) (Manjón et al., 2013, 2015) to more objective (MPPCA) approaches (Ding et al., 2010; Veraart, Fieremans, & Novikov, 2016; Veraart, Novikov, et al., 2016). The objectivity of the latter approach is a great advantage, ensuring that only noise is removed while signal components are untouched, thereby leading to data that are denoised but not smoothed or otherwise corrupted. The existing recommendations for the application of MPPCA denoising already acknowledge the issue of spatial correlations and suggest applying the denoising routing at the earliest step of signal reconstruction and processing (Ades-Aron et al., 2020; Does et al., 2019; Fernandes et al., 2022; Moeller et al., 2021; Mosso et al., 2022; Olesen et al., 2023; Simões et al., 2022; Veraart, Novikov, et al., 2016; Vizioli et al., 2021). However, in practice, this is seldom possible, whether due to the vendor’s data output format (e.g., coil-combined magnitude images) and/or due to the relative difficulty in executing a complex image reconstruction pipeline for many users. As shown explicitly in this study, correlations can exist in reconstructed datasets—whether due to commonly used partial Fourier encoding, interpolation, or gridding of data sampled in a non-cartesian way—and, as shown here (and anticipated in previous work), they can significantly degrade the performance of the MPPCA algorithm. Hence, in this study, we sought to develop and explore PCA denoising approaches that increase the robustness of the classification of components containing mostly noise or signal to the effects of spatially correlated noise. Using an additional explicit measurement of the noise variance (i.e., GPCA and TPCA denoising), our results suggest that the deleterious effects of spatial correlations can be mitigated significantly.

### 4.1 Improved GPCA and TPCA performance over MPPCA

As expected, when spatial correlations are negligible, the novel denoising procedures described here have identical performance to MPPCA denoising (e.g., Fig. 1). However, when spatial correlations are introduced, our simulations and experiments clearly showed that GPCA and TPCA outperform MPPCA (Fig. 2 and Fig. 3A-B). The reason for the compromised MPPCA performance is that ${\hat{\sigma }}_{MP}^{2}$ is overestimated (Fig. 2F-G) as an effect of the increased eigenvalue spectral width in the presence of spatially correlated noise (Fig. 2H); consequently, the criterion for MPPCA component classification ${\bar{\lambda }}_{c}\geq {\hat{\sigma }}_{MP}^{2}$ is met only when just a few components are considered as noise carrying (cyan solid vertical line in Fig. 2F-G). GPCA on the other hand, since ${\bar{\lambda }}_{c}$ is unaffected by spatial correlations (c.f. derivations in Supplementary Material Appendix A, which show that ${\bar{\lambda }}_{c}$ is an unbiased estimator of the noise variance for arbitrary uncorrelated/correlated noise eigenvalue spectrum distribution), ${\bar{\lambda }}_{c}\leq {\hat{\sigma }}_{prior}^{2}$ is met as long as ${\hat{\sigma }}_{prior}^{2}$ is accurately estimated (green dashed vertical line in Fig. 2F-G). For TPCA, ${\hat{\sigma }}_{max}^{2}=\max\left(\lambda_{c}\right)/{\left(1+\sqrt{\gamma }\right)}^{2}$ is increased by spatially correlated noise but since the procedure compares ${\hat{\sigma }}_{max}^{2}$ to ${\hat{\sigma }}_{prior}^{2}$, the criterion ${\hat{\sigma }}_{max}^{2}<{\hat{\sigma }}_{prior}^{2}$ is satisfied when only a few components carrying mostly noise are misclassified (orange dashed vertical line in Fig. 2F-G), and thus still providing good denoising performance (Fig. 2E).

The limitations of the MPPCA denoising are very clearly noticeable in the pre-clinical data (Fig. 4B and Fig. 5), where the introduction of spatially correlated noise is coupled to an accurate estimation of the noise variance. Therefore, both GPCA and TPCA successfully overcome the MPPCA limitations. In the human data, MPPCA also performs sub-optimally (Fig. 6) as highlighted by the larger number of PCA components preserved (Fig. 6C1 and Fig. 6D) and by deviations between sampled residual quantiles and the theoretical Gaussian quantiles (Fig. 7F). However, since here the noise variance is more difficult to estimate, TPCA provides a better compromise between noise removal and signal preservation. The better performance of GPCA/TPCA denoising over MPPCA was observed directly in diffusion-weighted images (Fig. 4 and Fig. 6), in parametric DKI maps (Fig. 5 and Fig. 7), and in enhanced quantification of high angular information from the high b-value dMRI data (Fig. 8).

Although for our main analysis, MPPCA denoising was implemented based on the moment-matching algorithm proposed by Veraart, Novikov, et al. (2016), in Supplementary Figure S9 we show that MPPCA denoising by directly fitting the MP distribution to the PCA eigenvalue spectrum (MPPCA-slow, Veraart, Fieremans, & Novikov, 2016) also classifies only a few components as mostly carrying noise when noise is spatially correlated. For this alternative MPPCA denoising algorithm, we notice that only a few noise components are classified due to the discrepancies between the measured eigenvalue spectrum shape and the expected ground-truth MP distribution (c.f. Supplementary Fig. S9, panels B4-5). Therefore, we show that the MPPCA limitations pointed here are general for both MPPCA moment-matching (Veraart, Novikov, et al., 2016) and MPPCA-slow algorithms (Veraart, Fieremans, & Novikov, 2016).

Taken together, these results illustrate the general benefits of GPCA and TPCA in suppressing noise for advanced diffusion MRI techniques. We expect that these findings can be generalized to other advanced dMRI signal representations, microstructural models, ODF, and tractography reconstructions.

### 4.2 Comparison between GPCA and TPCA denoising

Theoretically, GPCA provides a more general eigenvalue classification criteria than MPPCA and TPCA, since ${\bar{\lambda }}_{c}$ is still a good proxy to the noise variance when components containing mostly noise are properly classified regardless of the specific assumption of the MP distributions (c.f. Supplementary Material Appendix A and Fig. 2), and thus, one may expect that GPCA would always produce more robust results. This clearly depends strongly on the quality of estimating ${\hat{\sigma }}_{prior}$. Indeed, when ${\hat{\sigma }}_{prior}$ estimates are unbiased, our simulations show that GPCA is the only technique that exactly classifies the correct number of signal and noise components (Fig. 2 and Fig. 3A-B).

However, our work also demonstrates that the performance of GPCA is more sensitive to ${\hat{\sigma }}_{prior}$ overestimations than the TPCA approach (Fig. 1I, Fig. 2I, Fig. 3C-D and Supplementary Fig. S6B). This can be explained by the different degrees that the inclusion of eigenvalues related to relevant signal contribution affects the different quantities used by GPCA and TPCA. Larger ${\hat{\sigma }}_{prior}$ overestimations are better tolerated by the TPCA classification criterion (${\hat{\sigma }}_{max}^{2}<{\hat{\sigma }}_{prior}^{2}$) than by the effect GPCA classification criterium (${\bar{\lambda }}_{c}>{\hat{\sigma }}_{prior}^{2}$)—note that a larger bias in ${\hat{\sigma }}_{max}^{2}$ (used by TPCA) than in ${\bar{\lambda }}_{c}$ (used by GPCA) is present when signal components are included (c.f. Figs. 1F-G and Figs. 2F-G).

Consequently, TPCA will be more robust to ${\hat{\sigma }}_{prior}^{2}$ misestimation. This observation is relevant for real-life experiments (especially for *in vivo* acquisitions in clinical scanners), since ${\hat{\sigma }}_{prior}$ estimation is more likely to be compromised by image artifacts. For instance, in our human diffusion MRI data, (Fig. 6A) ${\hat{\sigma }}_{prior}$ is very likely overestimated, and thus, when GPCA is used, it clearly removes true signal components as indicated by the smaller number of signal components near the regions with higher ${\hat{\sigma }}_{prior}$ estimates (Fig. 6C3). In the diffusion parametric maps, the removal of true signal components was associated with the corruption of diffusion anisotropy as revealed in both DKI FA maps (Fig. 7) and Q-ball GFA maps (Fig. 8), which is in line with the predictions from simulations (Fig. 3C-D). Simulations also revealed that fiber direction estimates may be compromised when overestimated ${\hat{\sigma }}_{prior}$ is used (Supplementary Fig. S6B). Hence, GPCA should be used mainly when ${\hat{\sigma }}_{prior}$ is robustly estimated—for example, in ex-vivo data where no motion/flow effects exist and where then repetitions can be used more robustly to determine the variance.

It is worth noting that the loss of signal due to GPCA denoising can be challenging to observe qualitatively through residual maps (Supplementary Fig. S8), which are commonly used for evaluating denoising performance in diffusion MRI. Our results emphasize the need for alternative methods of analysis, such as assessing the impact on diffusion parametric maps that capture non-Gaussian and anisotropic diffusion effects or using residual normality tests to assess denoising performance.

### 4.3 Relevance to other recent PCA denoising advances

To improve the robustness of MPPCA denoising in non-central distributed, spatially varying, and correlated noise, recent studies proposed the use of complex MRI data in which noise can be approximately characterized by a Gaussian distribution (Cordero-Grande et al., 2019; Moeller et al., 2021; Vizioli et al., 2021). Using the information in complex data, these procedures also apply additional pre-processing steps to minimize noise correlations from parallel imaging reconstructions and to uniformize the spatial noise variation. In the present study, these additional pre-processing steps prior data denoising were not considered since we were interested in exploring strategies applicable to typical magnitude reconstructed data. Therefore, we focused on the effects of spatially correlated noise and explored how different PCA threshold criteria for signal and noise component classification can still provide robust denoising performance of magnitude reconstructed MRI data. For this, the need for correcting spatially varying noise in GPCA and TPCA is bypassed by using local noise level estimation, while correction of non-central distributed noise of magnitude data is ignored assuming that SNR is sufficiently high in most imaging voxels (more details on this are discussed below, see section 4.4).

It is important to note that, in addition to the current study, alternative PCA threshold criteria for signal and noise component classification were also considered by the Noise Reduction with Distribution Corrected (NORDIC) PCA method (Moeller et al., 2021; Vizioli et al., 2021), in which an upper bound for thresholding components containing mostly noise is computed with Monte-Carlo simulations of complex noisy matrices with a prior variance estimate. As mentioned in section 2.1.4, this approach is very similar to TPCA which also uses noise variance estimates to compute the noise eigenvalue upper band. TPCA uses, however, the analytical solution provided by the MP distribution (Eq. 9) instead of resorting to Monte-Carlo simulations. Although TPCA avoids stochastic errors from Monte-Carlo simulations, due to their similar nature, the assessment of TPCA in this study is also representative of the NORDIC technique when excluding the spatial noise variation homogenization steps for compatibility to process magnitude data.

### 4.4 Limitations and future work

To illustrate the poor performance of MPPCA on data with high spatially correlated noise, we only used partial Fourier data reconstructed using zero-filling. However, it is important to note that the use of more advanced procedures to reconstruct partial Fourier data may mitigate the effects of spatially correlated noise. In this study, more advanced data reconstruction procedures are not considered since our aim was to test PCA denoising strategies in data exhibiting highly correlated noise and since zero-filling data reconstruction is still nowadays a common approach adopted in the reconstruction procedures for both pre-clinical and clinical scanners.

As mentioned above, the “cost” of the new GPCA and TPCA denoising approaches is the need to estimate the noise variance a-priori. In many cases (such as ours), this information can be obtained from just a few repeated b = 0 images, which are quite commonly acquired in conventional datasets. This approach has the advantage of computing the effective noise variance after image reconstruction, avoiding strong assumptions on how noise spatially varies across adjacent voxels (Constantinides et al., 1997; Landman, Bazin, & Prince, 2009; Landman, Bazin, Smith, et al., 2009; Sodickson et al., 1999). Although in this study we used a relatively large number of b = 0 image repetitions to compute the noise variance prior (20 for the pre-clinical data and 5 for the clinical dataset), Supplementary Figure S1 shows that GPCA and TPCA can still be used for just 2 b-value repetitions at the price of having less precise ${\hat{\sigma }}_{prior}^{2}$ estimates, which, when used by GPCA and TPCA, will induce only a slightly increase of the number of preserved components. In this scenario however, TPCA and GPCA still outperform MPPCA when the noise is spatially correlated (Supplementary Fig. S1).

In addition to ${\hat{\sigma }}_{prior}$ precision issues, noise variance estimates obtained from repeated images can also be sensitive to artifacts which can bias ${\hat{\sigma }}_{prior}$ and compromise the correct classification of signal components by our denoising strategies, particularly by GPCA (as discussed above). In this study, these biases are minimized by taking the median of ${\hat{\sigma }}_{prior}$ values across the voxels for each sliding window instance. However, in future studies, the exploration of alternative noise prior estimation strategies (e.g., Aja-Fernández et al., 2009, 2015; Landman, Bazin, & Prince, 2009; Landman, Bazin, Smith, et al., 2009; Liu et al., 2014; Pieciak et al., 2017; Tabelow et al., 2015) could be of interest to promote the general use of GPCA and TPCA procedures in different multidimensional MRI datasets.

As mentioned above, in this study, pre-processing steps to correct for spatially varying noise prior to denoising as done by Moeller et al. (2021) and Vizioli et al. (2021) are not considered. Instead, spatially varying noise is considered by using spatially varying noise estimates (c.f. Fig. 4A1, Fig. 4B1, Fig. 6A3). Although this provides a simple way to deal with spatially varying noise in magnitude reconstructed data, the use of these noise maps is only appropriate if noise varies slowly across the image so that noise can be assumed to be relatively uniform across the sliding window. In practice, when spatially varying noise is observed (as the case of our clinical acquisitions, c.f. Fig. 6A2-3), this assumption may not be satisfied for large sliding windows. In this case, reducing the dimensions of the sliding window must be balanced against the need for redundancy.

Here, our proposed methods are intentionally applied without incorporating any correction for the non-central distributed noise to allow the assessment of the robustness of PCA denoising algorithms without imposing prior pre-processing steps that necessitate information from complex data. Although our PCA denoising strategies assume identically distributed noise (assumption that is violated by non-central distributed noise), GPCA and TPCA were shown to perform well even on magnitude data in which noise is non-central distributed. This is also supported by Supplementary Figure S3 in which the performance of GPCA and TPCA was stable even in phantoms corrupted by synthetic spatially correlated Rician noise. It is important to note that although in this work we focus on denoising magnitude data, the eigenvalue classification criteria in GPCA and TPCA applies for complex MRI data. Therefore, in future studies, GPCA and TPCA can be integrated to other advanced denoising routines that use the complex data to ensure zero-mean distributed noise, homogenization of spatially varying noise, phase stabilization (Bazin et al., 2019; Moeller et al., 2021; Vizioli et al., 2021), higher dimensional MRI data (Olesen et al., 2023), or into multi-coil image reconstruction procedures (Lemberskiy et al., 2021). For example, inserting TPCA into NORDIC could be advantageous to avoid any stochastic error of Monte-Carlo based approaches. Additionally, future studies could compare our approaches with other denoising strategies that have been emerging in the last years, for example, algorithms based on machine-learning procedures such as Fadnavis et al. (2020) and Muckley et al. (2021). These comparisons should be explored in dedicated studies due to the different nature of advantages and disadvantages across the different classes of denoising algorithms.

## 5 Conclusion

The impact of spatially correlated noise on the performance of PCA denoising was evaluated and found to corrupt the performance of the commonly used MPPCA denoising algorithm, especially when data are acquired with Partial Fourier and reconstructed by modern schemes. We proposed two new PCA strategies (GPCA and TPCA) that harness prior information on noise variance estimates to objectively denoise MRI data contaminated by spatially correlated noise. Our work shows that both GPCA and TPCA denoising can enhance the quality of diffusion maps and orientation distribution function estimates in both pre-clinical and clinical settings. TPCA is more robust from a practical perspective in clinical data since it is more immune to noise variance overestimations, which are common in clinical settings. Both GPCA and TPCA denoising algorithms are readily generalizable and can therefore be used for denoising other types of redundant data, thereby enabling higher resolution and better overall performance in MRI.

## Supplementary Material

Supplementary Material

## Data Availability

Data used for this study are available at https://zenodo.org/records/10200469. All code and analysis scripts are available at https://github.com/RafaelNH/PCAdenoising.
