# Supplementary material for: Efficient PCA denoising of spatially correlated redundant MRI data
Source: Imaging Neurosci (Camb). 2023 Dec 18;1:imag-1-00049. doi: 10.1162/imag_a_00049 (PMC12180759; doi:10.1162/imag_a_00049)
Supplement: Supplementary Material [file imag_a_00049-supp.pdf]

Efficient PCA denoising of spatially correlated redundant MRI data

Supplementary Material

Rafael Neto Henriques[1], Andrada Ianuş[1], Lisa Novello[4], Jorge Jovicich[4], Sune N Jespersen[2,3], Noam Shemesh[1*]

[1]*Champalimaud Research, Champalimaud Foundation, Lisbon, Portugal*
[2]*Center of Functionally Integrative Neuroscience (CFIN) and MINDLab, Clinical Institute, Aarhus University, Aarhus, Denmark.*
[3]*Department of Physics and Astronomy, Aarhus University, Aarhus, Denmark*
[4]*Center for Mind/Brain Sciences - CIMeC, University of Trento, Rovereto, Italy*

*Corresponding author:
Dr. Noam Shemesh, Champalimaud Research, Champalimaud Foundation, Av. Brasilia 1400-038, Lisbon, Portugal
E-mail: noam.shemesh@neuro.fchampalimaud.org
Phone number: +351 210 480 000 ext. #4467

Appendix A – General PCA Denoising Eigenvalue Classification

Consider a $M \times N$ matrix $\boldsymbol{X}$ that contains only noise with $\langle X_{ij} \rangle = 0$, $\langle X_{ij}^2 \rangle = \sigma^2$, that $\langle X_{ij} X_{kl} \rangle$ can be non-zero even for $(ij) \neq (kl)$, and $M \geq N$, then

$$T = \frac{1}{N} \boldsymbol{X}^T \boldsymbol{X} \Rightarrow$$

$$\langle \frac{1}{N} Tr(T) \rangle = \langle \frac{1}{N} \sum_{i=1}^{N} \lambda_i \rangle = \langle \lambda_i \rangle \Rightarrow$$

$$\langle \frac{1}{N^2} Tr(\boldsymbol{X}^T \boldsymbol{X}) \rangle = \langle \frac{1}{N^2} \sum_{i=1}^{N} (\boldsymbol{X}^T \boldsymbol{X})_{ii}) \rangle = \frac{1}{N^2} \sum_{i,k=1}^{N} \langle X_{ik} X_{ik} \rangle = \frac{1}{N^2} \sum_{i,k=1}^{N} \sigma^2 = \sigma^2$$

Therefore, $\langle \lambda_i \rangle = \sigma^2$ when matrix $\boldsymbol{X}$ contains only noise. When signal components are present $\langle \lambda_i \rangle > \sigma^2$, thus, the eigenvalue classification criterion for General PCA denoising consists in finding the larger number of eigenvalues that satisfies the following inequality:

$$\langle \lambda_c \rangle = \overline{\lambda_c} < \sigma^2$$

Note, that in contrast with the Marčenko-Pastur distribution, here, we do not assume that matrix $\boldsymbol{X}$ has entries with a probability distribution of finite fourth order, and that M and $N \rightarrow \infty$.

Appendix B – Simulation Supplementary Figures

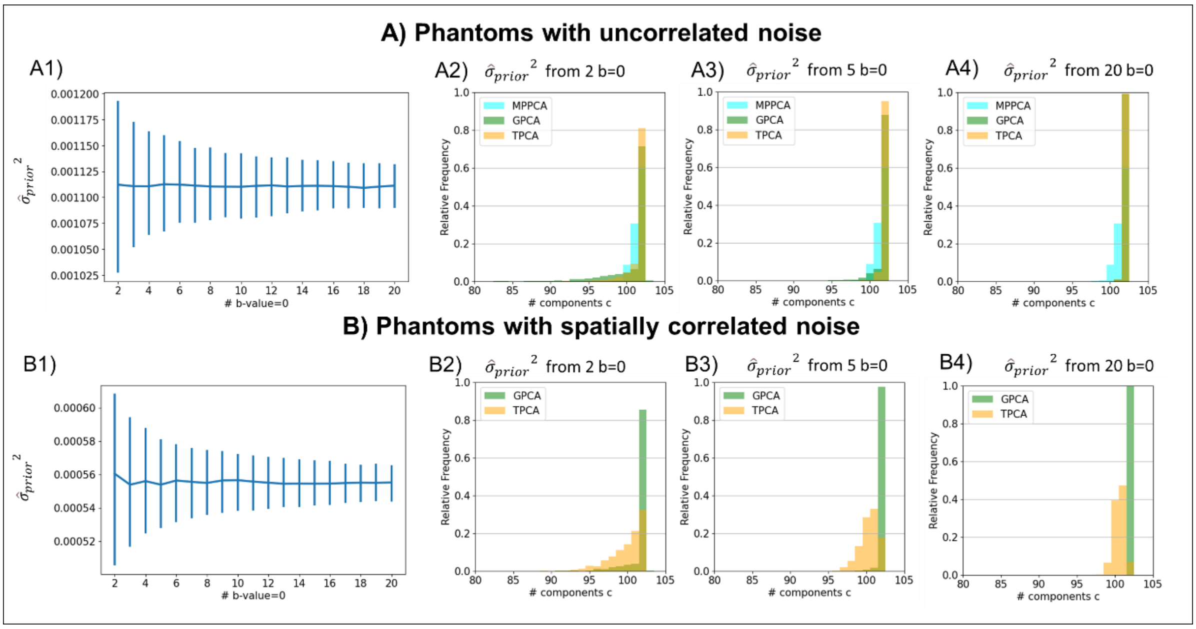

Supplementary Fig. S1 – Assessment of noise variance precision as a function of number of b-value=0 repetitions by running phantoms with uncorrelated (A) and with spatially correlated noise (B) for 1000 different instances. Median and interquartile ranges for noise variance estimates across all 1000 phantom instances computed for different b-value=0 signals (A1/B1) Relative frequencies of the number of noise component classified by the different denoising strategies (MPPCA/GPCA/TPCA) across the 1000 simulations instances when noise variance is estimated from 2 b-value=0 signals (A2/B2), 5 b-value=0 signals (A3/B3), and 20 b-value=0 signals (A4/B4). This supplementary figure shows that, unlike the MPPCA algorithm, GPCA and TPCA always classifies numbers of signal components near to the ground truth value of 102 when noise is spatially correlated. Note that relative frequencies for the moment-matching MPPCA algorithm were not presented in panels B2-4 since this always classified less than 80 noise components for all simulations instances.

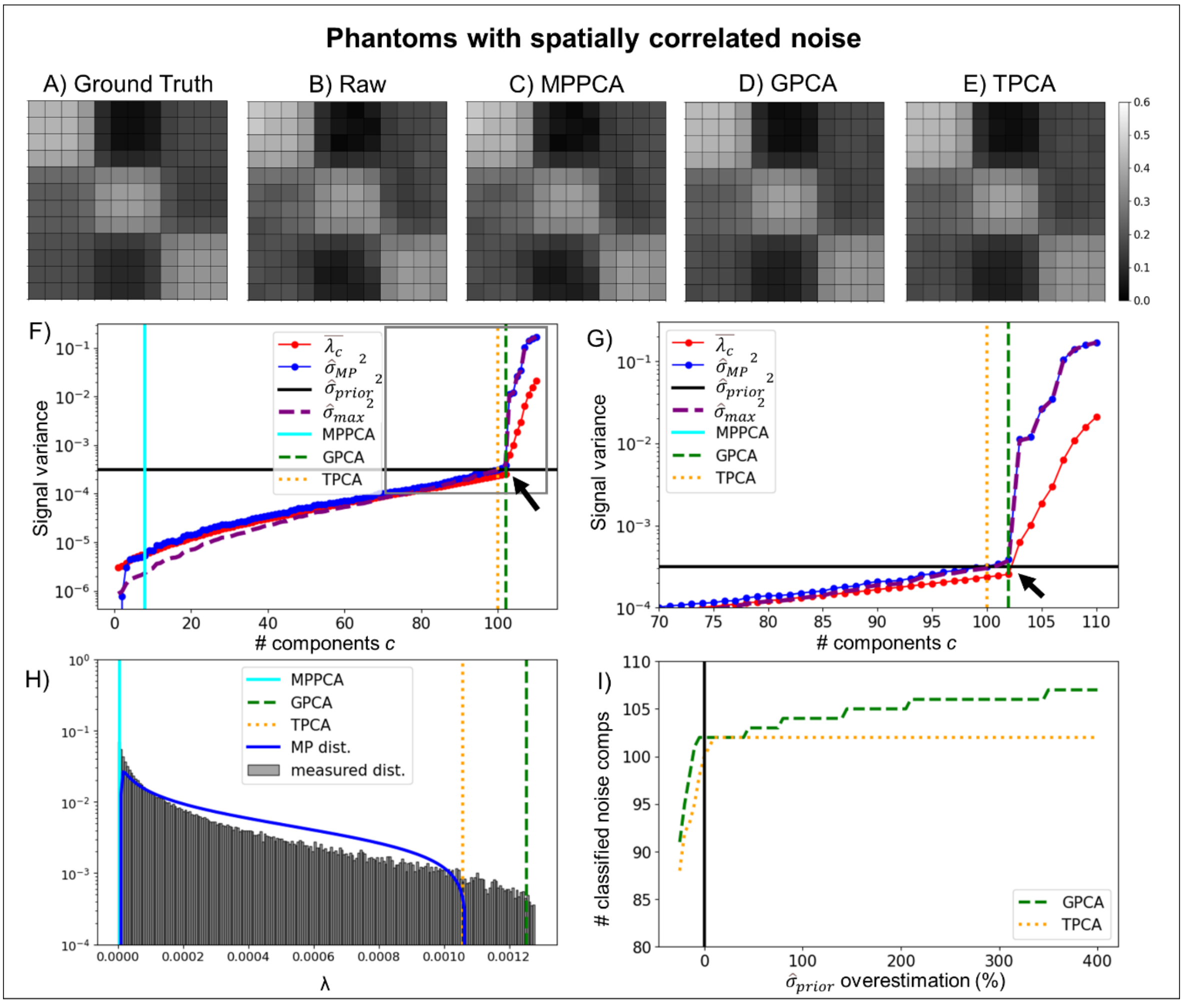

Supplementary Fig. S2 – Denoising performance in phantom simulation with spatially correlated noise generated by Smoothing data with a 2D Gaussian kernel with a standard deviation of 0.6 (i.e. 34% of signal and noise arising from neigbor voxels). Representative ground truth noise free **(A)** and noise corrupted **(B)** signals for a selected diffusion gradient direction of the highest diffusion gradient intensity alongside denoised signals for the MPPCA **(C)**, GPCA **(D)**, and TPCA **(E)** denoising algorithms. **(F)** Parameters assessed by the denoising algorithms plotted as a function of the number of lower eigenvalues potentially considered as noise. Thresholds for the MPPCA, GPCA and TPCA are plotted by the cyan solid, green dashed, and orange vertical lines respectively (black arrow point to the ground truth number of signal components, i.e., 102). **(G)** Zoomed plot of the parameters assessed by the denoising algorithms. **(H)** Reconstructed eigenvalue spectrum for 1000 trials and respective theoretical MP distribution for identical eigenvalue variances are shown in panel – the median thresholds for the MPPCA, GPCA and TPCA computed as the threshold median across the 1000 repetitions are plotted by the cyan solid, green dashed, and orange vertical lines respectively. **(I)** The number of classified noise components for both GPCA and TPCA as a function of the percentage overestimation of noise standard deviation is shown in panels. In general, this figure shows that the GPCA and TPCA denoising algorithms outperform MPPCA denoising even for phantom generated with a different type of spatially correlated noise than the one considered for the main simulations performed for this study.

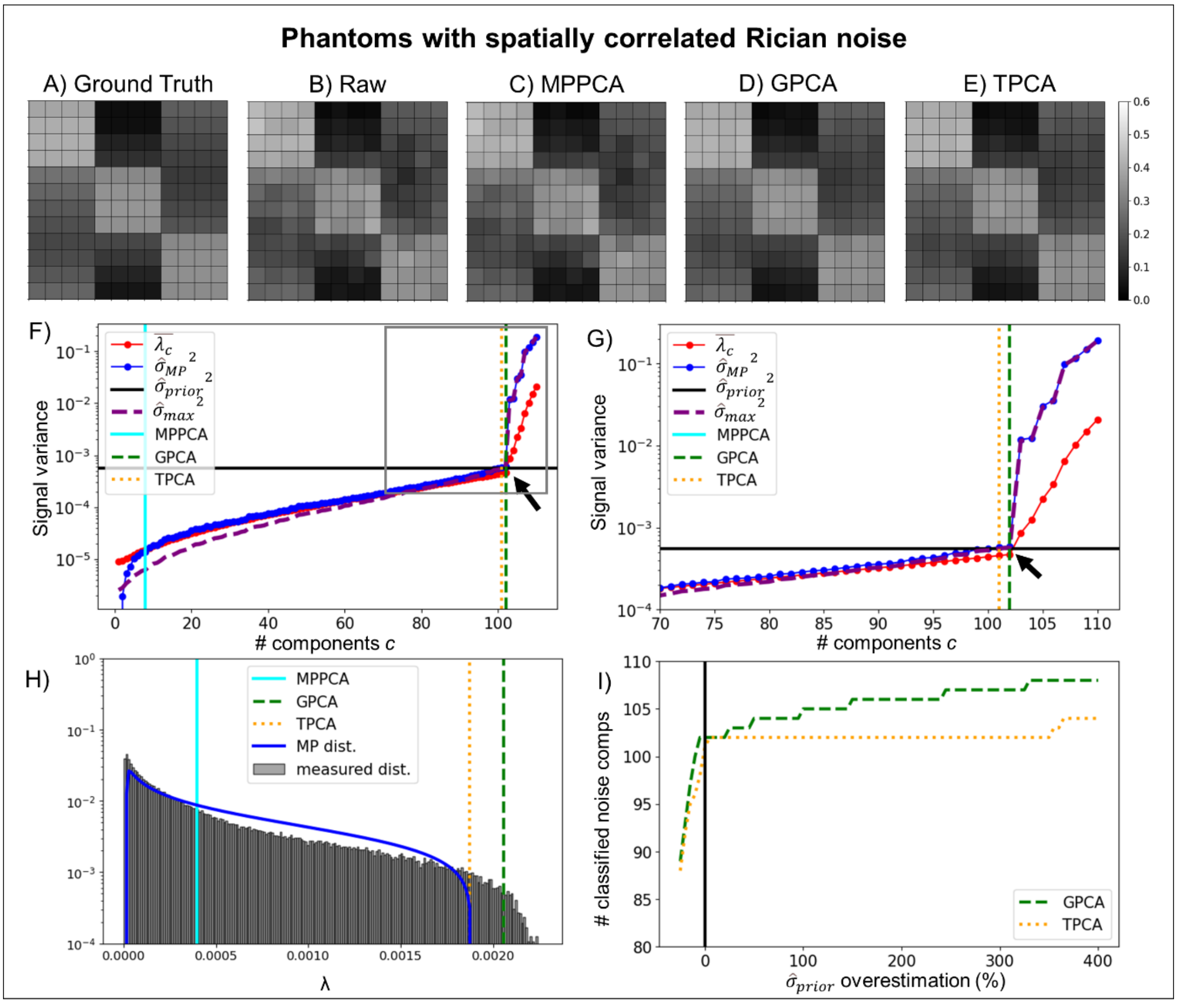

Supplementary Fig. S3 – Simulations of denoising performance in a phantom with correlated Rician noise - correlations were induced by zero filling in k-space. Representative ground truth noise free **(A)** and noise corrupted **(B)** signals for a selected diffusion gradient direction of the highest diffusion gradient intensity alongside denoised signals for the MPPCA **(C)**, GPCA **(D)**, and TPCA **(E)** denoising algorithms. **(F)** Parameters assessed by the denoising algorithms plotted as a function of the number of lower eigenvalues potentially considered as noise. Thresholds for the MPPCA, GPCA and TPCA are plotted by the cyan solid, green dashed, and orange vertical lines respectively (black arrow point to the ground truth number of signal components, i.e., 102). **(G)** Zoomed plot of the parameters assessed by the denoising algorithms. **(H)** Reconstructed eigenvalue spectrum for 1000 trials and respective theoretical MP distribution for identical eigenvalue variances are shown in panel – the median thresholds for the MPPCA, GPCA and TPCA computed as the threshold median across the 1000 repetitions are plotted by the cyan solid, green dashed, and orange vertical lines respectively. **(I)** The number of classified noise components for both GPCA and TPCA as a function of the percentage overestimation of noise standard deviation is shown in panels. Thus, GPCA and TPCA denoising algorithms outperform MPPCA denoising when noise even for spatially correlated Rician noise.

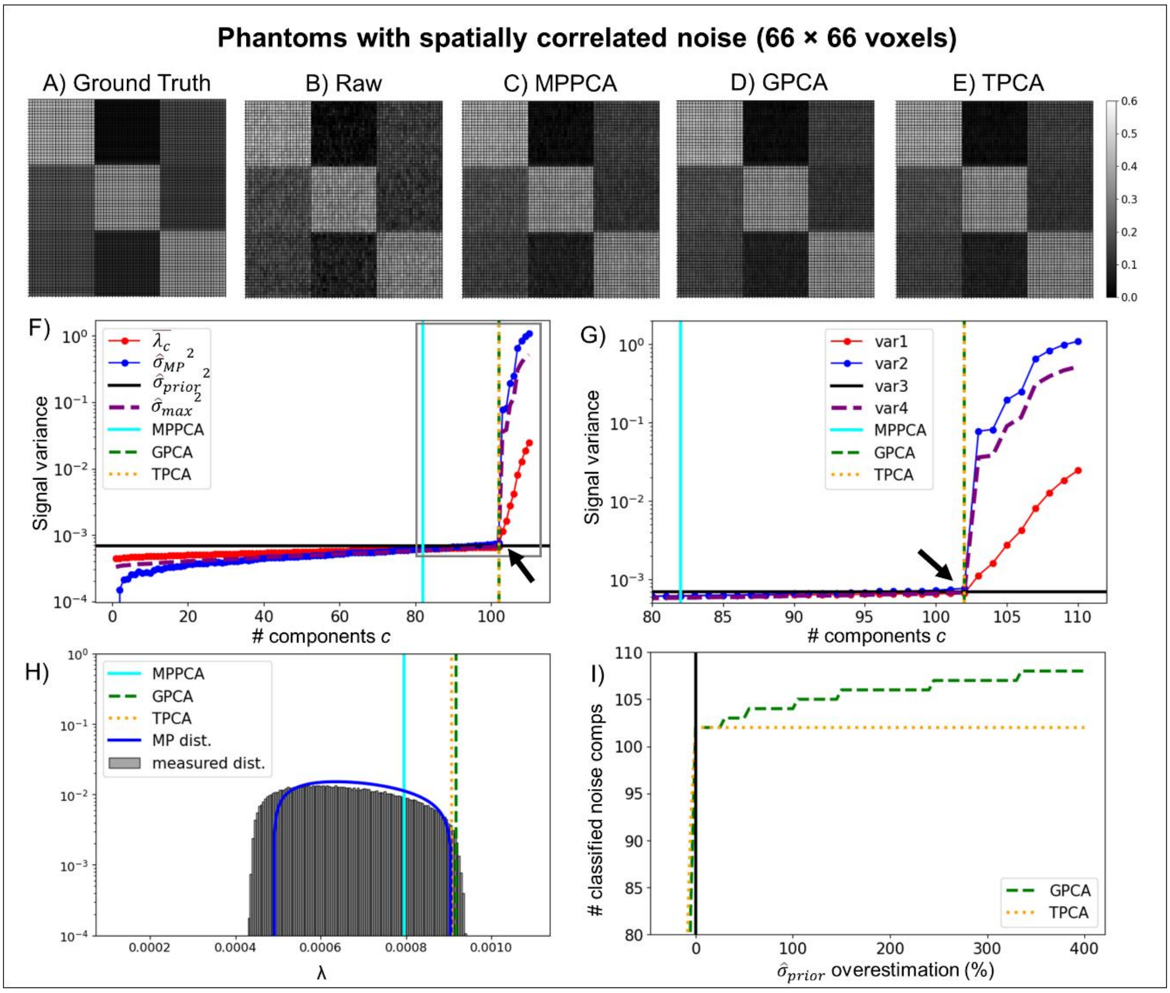

Supplementary Fig. S4 – Simulations of denoising performance in a phantom of 66 × 66 voxels with correlated noise. Representative ground truth noise free **(A)** and noise corrupted **(B)** signals for a selected diffusion gradient direction of the highest diffusion gradient intensity alongside denoised signals for the MPPCA **(C)**, GPCA **(D)**, and TPCA **(E)** denoising algorithms. **(F)** Parameters assessed by the denoising algorithms plotted as a function of the number of lower eigenvalues potentially considered as noise. Thresholds for the MPPCA, GPCA and TPCA are plotted by the cyan solid, green dashed, and orange vertical lines respectively (black arrow point to the ground truth number of signal components, i.e., 102). **(G)** Zoomed plot of the parameters assessed by the denoising algorithms. **(H)** Reconstructed eigenvalue spectrum for 1000 trials and respective theoretical MP distribution for identical eigenvalue variances are shown in panel – the median thresholds for the MPPCA, GPCA and TPCA computed as the threshold median across the 1000 repetitions are plotted by the cyan solid, green dashed, and orange vertical lines respectively. **(I)** The number of classified noise components for both GPCA and TPCA as a function of the percentage overestimation of noise standard deviation is shown in panels. Thus, GPCA and TPCA denoising algorithms outperform MPPCA denoising when noise is spatially correlated even for larger phantom sizes.

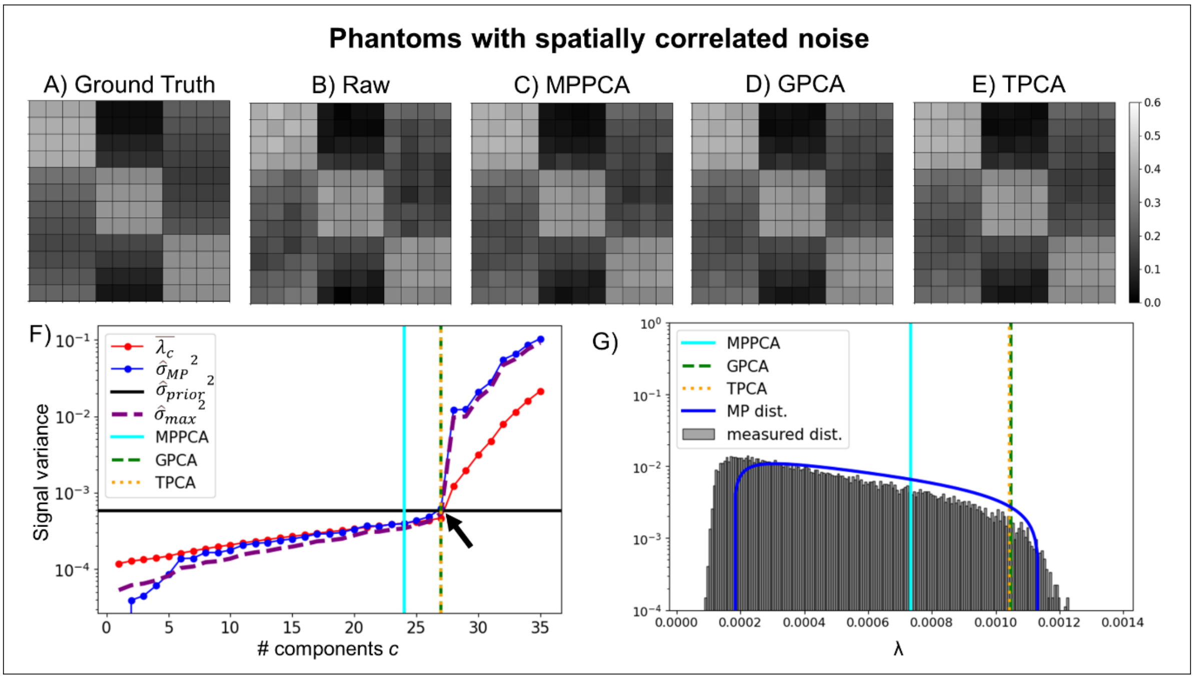

Supplementary Fig. S5 - Denoising performance in phantoms with a smaller number of diffusion MRI experiments (30 directions for b-value = 2 ms/μm^2 + 5 b-value=0 signals) with spatially correlated noise (zero-filling). Representative ground truth noise free **(A)** and noise corrupted **(B)** signals for a selected diffusion gradient direction of the highest diffusion gradient intensity alongside denoised signals for the MPPCA **(C)**, GPCA **(D)**, and TPCA **(E)** denoising algorithms. **(F)** Parameters assessed by the denoising algorithms plotted as a function of the number of lower eigenvalues potentially considered as noise. Thresholds for the MPPCA, GPCA and TPCA are plotted by the cyan solid, green dashed, and orange vertical lines respectively (black arrow point to the ground truth number of signal components, i.e., 35 total components – 8 signal components = 27 noise components). **(G)** Reconstructed eigenvalue spectrum for 1000 trials and respective theoretical MP distribution for identical eigenvalue variances are shown in panel – the median thresholds for the MPPCA, GPCA and TPCA computed as the threshold median across the 1000 repetitions are plotted by the cyan solid, green dashed, and orange vertical lines respectively. Thus, GPCA and TPCA denoising algorithms outperform MPPCA denoising for phantoms generated for a smaller number of diffusion MRI experiments. Note that the noise reduction in panels D-E are not so evident as the noise reduction observed in Fig. 1 and Fig. 2; however, this can be explained by the lower SNR gains expected when data contains a smaller number of redundant MRI acquisitions.

Supplementary Table S1 - Root-mean-squared-error for DKI estimates extracted from raw and denoised phantoms corrupted by spatially correlated data (zero-filling).

	Unbiased $\hat{\sigma}_{prior}$				overestimated $\hat{\sigma}_{prior}$ (200%)	
	Raw	MPPCA	GPCA	TPCA	GPCA	TPCA
**FA**	0.0181	0.0170	0.0103	0.0109	0.0184	0.0101
**MD** (μm^2/ms)	0.0171	0.0157	0.0075	0.0083	0.0174	0.0075
**RD** (μm^2/ms)	0.0157	0.0146	0.0080	0.0086	0.0158	0.0079
**AD** (μm^2/ms)	0.0510	0.0477	0.0273	0.0292	0.0477	0.0269
**MK**	0.0758	0.0688	0.0323	0.0354	0.0541	0.0327
**RK**	0.2811	0.2598	0.1533	0.1640	0.8221	0.1510
**AK**	0.2558	0.2440	0.1976	0.2069	0.3180	0.1941

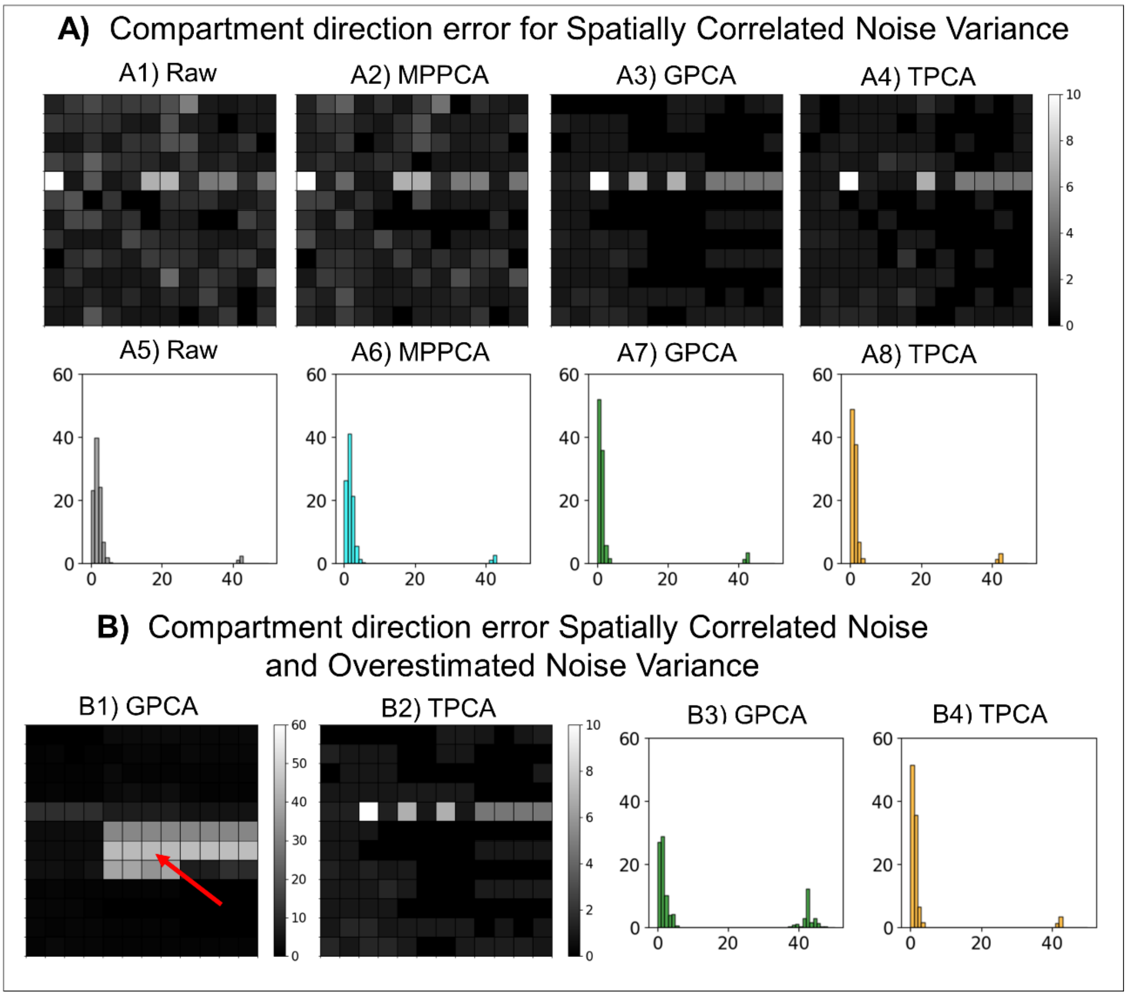

Supplementary Fig. S6 – Simulated denoising performance in compartment's direction estimates. Direction estimates are obtained by extracted from Q-ball orientation distribution function (ODF) reconstructions using the highest b-value signals (together with b-value=0 signal repetitions). Angular errors are computed for the larger ODF peak relative to the nearest ground truth direction. In panels A, upper panels show the angular error maps (in degrees) computed from noise corrupted (A1, B1), MPPCA denoised (A2, B2), GPCA denoised (A3, B3), and TPCA denoised (A4, B4) signals, while lower panels show the angular error histograms computed by repeating 100 noise instances (corresponding to a total of 14400 = 144 voxels per phantom × 100 noisy phantom instances) for raw and all four denoising strategies. In panels B, upper panels show the angular error maps computed from GPCA (B1) and TPCA (B2) denoised signals and respective residuals histograms (B3, B4) when overestimated noise variance (200% of its original value) is used. Red arrow in panels B1, indicates regions that direction estimates are highly compromised. This figure shows that TPCA and GPCA preserves compartment direction estimates when the noise variance is accurately estimated; however, as for DKI parametric maps, GPCA can corrupt directional information when noise is overestimated.

Appendix C – Pre-clinical Data Supplementary Figures

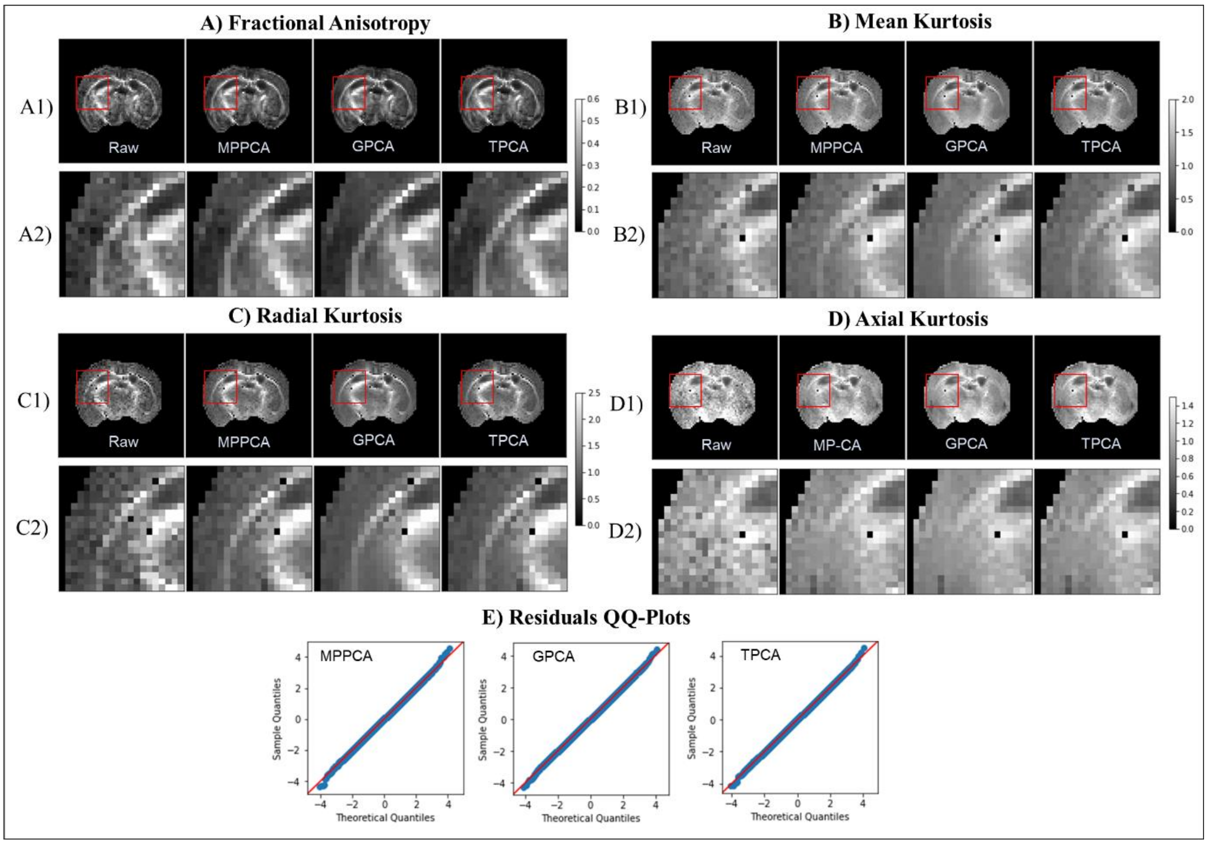

Supplementary Fig. S7 – DKI maps for the pre-clinical dataset acquired to minimize the effects of spatially correlated noise and QQ-plots of the denoising residuals: A) Fractional Anisotropy; B) Mean Kurtosis; C) Radial Kurtosis; D) Axial Kurtosis; E) QQ-plots of the denoising residuals. For each DKI quantity, images are displayed for an entire representative axial slice (A1, B1, C1, D1) and for the zoomed area marked by the red box (A2, B2, C2, D2). From left to right, DKI maps are displayed for the raw, MP-PCA denoised, GPCA denoised and TPCA denoised data. QQ-plots of the denoising residuals for the selected zoomed region are show panel E. This supplementary figure shows that, for data acquired with minimized noise spatial correlations, all denoising procedures (MP-PCA, GPCA, TPCA) improves DKI map estimation in a similar fashion. In this case, residual distributions for all denoising techniques is close to a theoretical Gaussian distribution.

Appendix D – Clinical Data Supplementary Figures

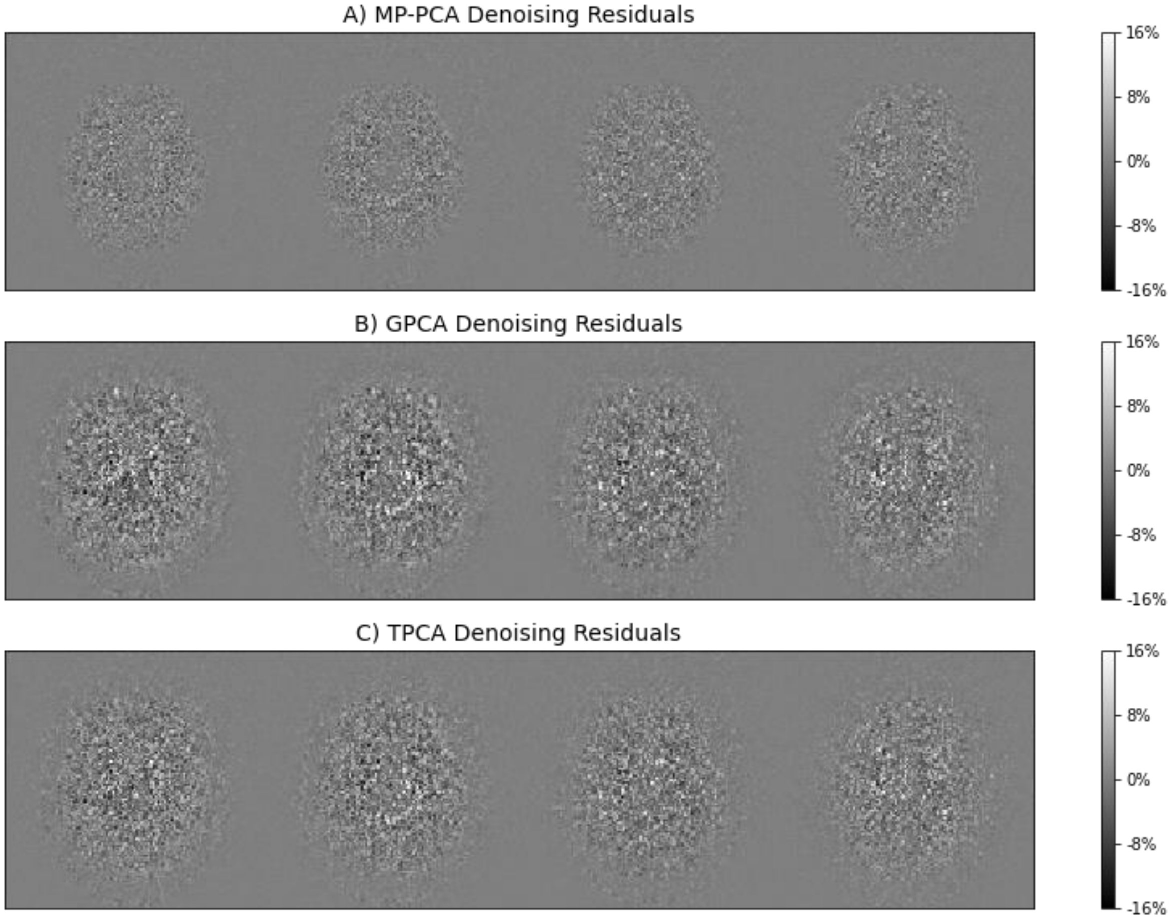

Supplementary Fig. S8 – Denoising residuals of the clinical dataset for the three different denoising procedures: A) MPPCA denoising; **B)** GPCA denoising; and C) TPCA denoising. Residuals are shown for the same representative slices of the main article Figure 6, i.e. residuals are shown for images acquired with gradient direction near v = [1, 0. 0] and for b-value = 2, 3, 4.5, and 6 ms/μm2 (from left to right). This supplementary figure was produced to show that it is hard to inspect loss of structural information on denoising residual maps.

Appendix E – Supplementary Figures for MPPCA-slow Assessment

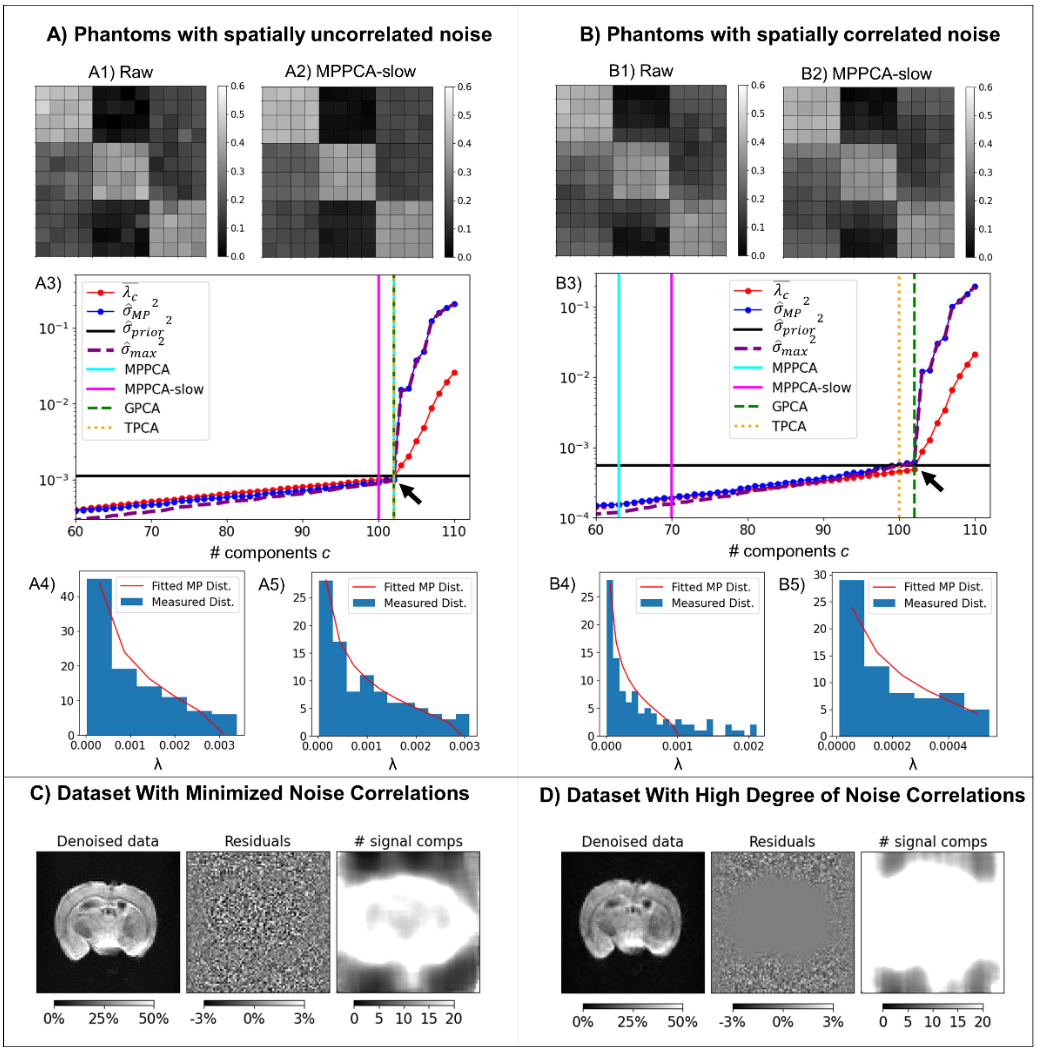

Supplementary Fig. S9 – MPPCA-slow performance in phantom with uncorrelated noise (A), phantom with zero-filling spatially correlated noise (B), preclinical dataset with minimized noise correlations (C), and preclinical dataset with high degree of noise correlations (D). Simulations in panels A and B correspond to the same simulations reported in Fig. 1 and 2, and its sub-panels show: (A1/B1) reference raw diffusion-weighted signals selected diffusion gradient direction of the highest diffusion gradient intensity; (A2/B2) denoised diffusion-weighed signals for the MPPCA-slow algorithm; (A3/B3) MPPCA-slow calculated threshold (vertical magenta line) plotted together with the parameters assessed by the other denoising algorithms (MPPCA, GPCA, TPCA which thresholds are plotted by the cyan solid, green dashed, and orange vertical lines respectively); (A4/B4) MP histograms (red) fitted to the measured eigenvalue spectrum (blue) when the 102 ground truth values are considered; and (A5/B5) MP histograms (red) fitted to the measured eigenvalue spectrum (blue) using the number of noise components calculated after MPPCA-slow convergence. Note that the MP histograms fitted to the eigenvalue spectrum of the 102-ground truth noise components only includes 85 noise components when noise is spatially correlated (panel B4), while the MP histogram fitted to the MPPCA-slow classified number of components only includes 70 components (i.e., MPPCA-slow tends to classify less and less noise components with it iteration progression). From left to right, panes C and B shows a sample MPPCA-slow denoised diffusion-weighted image acquired with b-value=3 ms/µm^2, its corresponding denoising residual map, and its preserved number of components map. In general, MPPCA-slow has a good denoising performance for simulations with uncorrelated noise and for data acquired to minimize noise spatial correlations; however, as the moment-matching MPPCA algorithm, MPPCA-slow fails to denoise data in the presence of spatially correlated noise.
